# Supplementary material for: Serious game versus standard care for rehabilitation after distal radius fractures: a protocol for a multicentre randomised controlled trial
Source: BMJ Open. 2021 Mar 30;11(3):e042629. doi: 10.1136/bmjopen-2020-042629 (PMC8030479; doi:10.1136/bmjopen-2020-042629)
Supplement: Author's manuscript [file bmjopen-2020-042629.draft_revisions.pdf]

BMJ Open

BMJ Open is committed to open peer review. As part of this commitment we make the peer review history of every article we publish publicly available.

When an article is published we post the peer reviewers' comments and the authors' responses online. We also post the versions of the paper that were used during peer review. These are the versions that the peer review comments apply to.

The versions of the paper that follow are the versions that were submitted during the peer review process. They are not the versions of record or the final published versions. They should not be cited or distributed as the published version of this manuscript.

BMJ Open is an open access journal and the full, final, typeset and author-corrected version of record of the manuscript is available on our site with no access controls, subscription charges or pay-per-view fees (http://bmjopen.bmj.com).

If you have any questions on BMJ Open's open peer review process please email info.bmjopen@bmj.com

BMJ Open

A serious game versus standard care for rehabilitation after distal radius fractures: a protocol for a multicentre randomised controlled trial

Journal:	*BMJ Open*
Manuscript ID	bmjopen-2020-042629
Article Type:	Protocol
Date Submitted by the Author:	09-Jul-2020

Complete List of Authors:	Meijer, Henriëtte; Amsterdam UMC Locatie AMC, Department of Surgery, Amsterdam Movement Sciences<br>Graafland, Maurits; Amsterdam UMC Locatie AMC, Department of Surgery<br>Obdeijn, Miryam; Amsterdam UMC Locatie AMC, Department of Plastic, Reconstructive and Hand Surgery<br>van Dieren, Susan; Amsterdam UMC Locatie AMC, Department of Surgery<br>Goslings, J. Carel; Onze Lieve Vrouwe Gasthuis, Department of Trauma Surgery<br>Schijven, Marlies; Amsterdam UMC Locatie AMC, Department of Surgery, Amsterdam Gastroenterology and Metabolism<br>ReValidate!, Collaborative research group; Amsterdam UMC Locatie AMC, Department of Surgery

Keywords:	Hand & wrist < ORTHOPAEDIC & TRAUMA SURGERY, REHABILITATION MEDICINE, Telemedicine < BIOTECHNOLOGY & BIOINFORMATICS, BIOTECHNOLOGY & BIOINFORMATICS, Information technology < BIOTECHNOLOGY & BIOINFORMATICS, Orthopaedic & trauma surgery < SURGERY

Note: The following files were submitted by the author for peer review, but cannot be converted to PDF. You must view these files (e.g. movies) online.
ReValidate! - Wrist fracture rehabilitation.wmv

SCHOLARONE™
Manuscripts

I, the Submitting Author has the right to grant and does grant on behalf of all authors of the Work (as defined in the below author licence), an exclusive licence and/or a non-exclusive licence for contributions from authors who are: i) UK Crown employees; ii) where BMJ has agreed a CC-BY licence shall apply, and/or iii) in accordance with the terms applicable for US Federal Government officers or employees acting as part of their official duties; on a worldwide, perpetual, irrevocable, royalty-free basis to BMJ Publishing Group Ltd ("BMJ") its licensees and where the relevant Journal is co-owned by BMJ to the co-owners of the Journal, to publish the Work in this journal and any other BMJ products and to exploit all rights, as set out in our licence.

The Submitting Author accepts and understands that any supply made under these terms is made by BMJ to the Submitting Author unless you are acting as an employee on behalf of your employer or a postgraduate student of an affiliated institution which is paying any applicable article publishing charge ("APC") for Open Access articles. Where the Submitting Author wishes to make the Work available on an Open Access basis (and intends to pay the relevant APC), the terms of reuse of such Open Access shall be governed by a Creative Commons licence – details of these licences and which Creative Commons licence will apply to this Work are set out in our licence referred to above.

Other than as permitted in any relevant BMJ Author's Self Archiving Policies, I confirm this Work has not been accepted for publication elsewhere, is not being considered for publication elsewhere and does not duplicate material already published. I confirm all authors consent to publication of this Work and authorise the granting of this licence.

For peer review only

A serious game versus standard care for rehabilitation after distal radius fractures: a protocol for a multicentre randomised controlled trial

H.A.W. Meijer, MD MSc[1], M. Graafland, MD PhD[2], M.C. Obdeijn, MD PhD[3], S. van Dieren, PhD[4], J.C. Goslings, MD PhD[5], M.P. Schijven, MD PhD MHSc[6], on behalf of the ReValidate!-collaborative study group

1: Department of Surgery, Amsterdam Movement Sciences, Amsterdam UMC, University of Amsterdam, Meibergdreef 9, P.O. Box 22660, 1100 DD Amsterdam, the Netherlands. Email: h.a.meijer@amsterdamumc.nl
2: Department of Surgery, Amsterdam UMC, University of Amsterdam, Meibergdreef 9, P.O. Box 22660, 1100 DD Amsterdam, the Netherlands. Email: m.graafland@amsterdamumc.nl
3: Department of Plastic, Reconstructive and Hand Surgery, Amsterdam UMC, University of Amsterdam, Meibergdreef 9, P.O. Box 22660, 1100 DD Amsterdam, the Netherlands. Email: m.c.obdeijn@amsterdamumc.nl
4: Department of Surgery, Amsterdam UMC, University of Amsterdam, Meibergdreef 9, P.O. Box 22660, 1100 DD Amsterdam, the Netherlands. Email: s.vandieren@amsterdamumc.nl
5: Department of Trauma Surgery, Onze Lieve Vrouwe Gasthuis, Jan Tooropstraat 164, P.O. Box 9243, 1006 AE Amsterdam, the Netherlands. Email: j.c.gosling@olvg.nl
6: Department of Surgery, Amsterdam Gastroenterology and Metabolism, Amsterdam UMC, University of Amsterdam, Meibergdreef 9, P.O. Box 22660, 1100 DD Amsterdam, the Netherlands. Email: m.p.schijven@amsterdamumc.nl

Corresponding author:

Prof. dr. M.P. Schijven, Department of Surgery, Amsterdam Gastroenterology and Metabolism, Amsterdam UMC, University of Amsterdam, Meibergdreef 9, P.O. Box 22660, 1100 DD Amsterdam, the Netherlands. Phone: +31 20 5664207. Email: m.p.schijven@amsterdamumc.nl

Word count:

Abstract: 248 words

Manuscript: 2970 words

For peer review only - http://bmjopen.bmj.com/site/about/guidelines.xhtml

ABSTRACT

Introduction

Distal radius fractures are amongst the most prevalent injuries worldwide. They are associated with high healthcare-related and socio-economic costs, mainly resulting from loss of productivity. To optimise recovery, wrist exercises are recommended. However, adherence to exercise regimens is low. Serious games provide a treatment platform for standardised postoperative care, uniting meaningful recovery with entertainment. Mobile serious games, including smartphone applications, are believed to improve self-efficacy.

Methods and analysis

To test effectiveness of a mobile serious game for distal radius fracture rehabilitation compared to standard care, a multicentre, randomised controlled trial was designed. Primary outcome will be the Patient-Rated Wrist Evaluation (PRWE) score after six weeks of treatment. Secondary outcomes are range of motion, grip strength, pain scores and self-reported treatment adherence.
Adult patients with any type of closed distal radius fracture are included. Patients are recruited in the outpatient clinics of four teaching hospitals. The intended sample size is 92 patients, based on the minimal clinically important difference of the PRWE score, using a superiority model.
Patients are randomised between using the wearable-controlled mobile serious game *ReValidate!* (intervention group), or standard care consisting of unsupervised exercises and a referral for physiotherapy upon request or recommendation by the treating clinician (control group).

Ethics and dissemination

The protocol has been approved by the Medical Ethical Review Board of the Academic Medical Centre in Amsterdam, the Netherlands. Results will be made available to healthcare providers, funders, and to the general public including patients via peer-reviewed academic journals and international conferences.

For peer review only

For peer review only - http://bmjopen.bmj.com/site/about/guidelines.xhtml

ARTICLE SUMMARY

Strengths and limitations of this study

- To the authors' knowledge, this randomised controlled trial comparing a wearable-controlled mobile serious game for wrist rehabilitation to standard care is the first of its kind, and may pave the way for future development of easily accessible digital rehabilitation games.
- The study is not only powered to show any statistically significant differences, but is sufficiently powered to show a clinically important difference in the outcome measurements of the primary endpoint, the patient-rated wrist evaluation score.
- Treatment adherence is measured by using a diary (subjective measurements) and by means of in-game registration (objective measurements), allowing for a reliable comparison between patient-reported and objectively monitored treatment adherence.
- The control group may receive heterogeneous treatment, yet this is an accurate representation of the current standard of care.
- The study staff and patients cannot be not blinded to the intervention due to the nature of the intervention, however the analysing statistician will be blinded to randomisation group.

Keywords:

Distal radius fracture; Rehabilitation; Physical therapy; e-Health; Serious game

Trial registration number: Dutch Trial Registry (NTR), NL6140, 12-04-2017, protocol version 2.

For peer review only

INTRODUCTION

Distal radius fractures are amongst the most frequently occurring traumatic injuries worldwide, occurring in 18% of patients that present to the Emergency Department after having sustained any fracture.[1,2] Up to 25% of fractures occurring in paediatric patients and 18% of fractures in the elderly population are distal radius fractures.[3] As the general population ages, the incidence of these injuries is increasing.[4-7] Not only are these injuries a burden to patients affected, they also present a significant socio-economic burden to society. Hand and wrist injuries are expensive mainly due to the loss of productivity. Distal radius fractures form the largest and most expensive group within this population.[8-10]

Distal radius fractures can be treated either operatively or conservatively.[11] In order to enhance recovery after initial fracture treatment, patients are often referred for physiotherapy or hand therapy. Yet, currently available scientific literature remains inconclusive on whether supervised physiotherapy or unsupervised exercise programmes should be advocated. [12-15] There is consensus that starting exercises early is preferred over starting later,[16-18] and performing any type of exercises is most likely more effective than performing no exercises.[19]

Similarly, surgical guidelines state that exercises are most likely beneficial.[20-22] These guidelines mostly leave it up to the clinician's preferences whether or not to refer a patient to a physiotherapist.[23] This leads to arbitrary rehabilitation regimens differing per region, hospital, and even per individual healthcare provider. It is currently unknown what percentage of patients follow-up to physiotherapy referrals. When patients are referred for physiotherapy and follow up to this referral, it has been found that only 19-35% of prescribed exercise regimens are executed completely and correctly.[24] Treatment adherence is influenced by practical constraints such as time, costs and travel distances.[25] Patients with a low self-efficacy will also perceive more barriers to treatment adherence.[26] Self-efficacy is defined as the belief one can be successful when performing a certain task, and is an important contributor to physiotherapy outcomes.[27,28]

For peer review only - http://bmjopen.bmj.com/site/about/guidelines.xhtml

In physiotherapy and rehabilitation, so-called 'serious games' have been gaining attention for their presumed effects on motivation and functional outcomes.[29-31] A serious game is defined as any kind of interactive computer application that incorporates gamification principles and serves an educational purpose, or aims to achieve a predefined goal.[32] Most studies evaluating the effect of serious games in physiotherapy, use consumer-based game consoles with an external hardware component, for example the Nintendo Wii™.[33-35] These types of games enable patients to perform rehabilitation exercises. As a downside, such games require a game-console and a monitor to function, limiting patients in their ability to exercise anywhere they want. A systematic review evaluating the use of serious games in rehabilitation of traumatic injuries described only 'off-the-shelf' games. No 'wearable-controlled' games could be identified.[31] Wearable sensors combined with easily accessible gaming platforms such as smartphones or tablets allow the user to exercise anytime and anywhere they want, and can decrease barriers to treatment adherence. To the best of our knowledge, there is no previous research on a home-based wearable-controlled serious game for traumatic injuries.

This multicentre, randomised controlled clinical trial aims to evaluate the effects on functional outcomes of a wearable-controlled serious game played on a smartphone or tablet, that is developed specifically for wrist rehabilitation. This is compared with the current standard treatment following distal radius fracture. This study hypothesizes that the use of the game will show a greater improvement in patient-rated functional outcomes compared to standard treatment. Secondary, this study aims to compare active range of motion, grip strength, treatment adherence, pain scores and to measure the effects on return to work between the two treatment strategies.

For peer review only - http://bmjopen.bmj.com/site/about/guidelines.xhtml

METHODS AND ANALYSIS

Study design

The study is a two-arm, parallel-group, multicentre randomised controlled superiority trial, designed to evaluate the effectiveness of a wearable-controlled serious game played on a smartphone or a tablet for distal radius fracture rehabilitation. Patients are randomised in a 1:1 ratio.

Participants and setting

Consecutive patients aged 18 years or older, with any type of conservatively or operatively treated closed distal radius fracture, are eligible to participate in the study. Patients are recruited from four different teaching hospitals in the country, of which one academic centre. Patients are recruited at the outpatient clinics, where clinicians will be approached for permission to contact their patients about the study. Before starting trial participation, written informed consent will be obtained from all participants by the research staff.

Patients with a medical history of loss of function due to injury or illness in either hand or wrist, polytraumatised patients, as well as patients that suffered bilateral wrist fractures or other injuries to the affected limb are excluded. Patients that are not in the possession of a smartphone or tablet compatible with the serious game, as well as patients with a visual or mental impairment preventing the proper use of smartphone or tablet are excluded. Those unable to understand spoken or written Dutch or English will be unable to complete the questionnaires and are therefore not eligible for participation.

Randomisation

When patients have been found eligible, and after they signed the informed consent form, baseline measurements are obtained. After baseline measurements, the local researcher contacts the central research coordinator for randomisation to take place, and to receive information on group allocation. Randomisation sequence is determined by an independent researcher using an online automatically

For peer review only

For peer review only - http://bmjopen.bmj.com/site/about/guidelines.xhtml

generated randomisation list (via www.sealedenvelope.com). Allocation concealment is obtained using sealed opaque envelopes. Patients are randomised using block randomization in blocks of four, stratified for age (18-64, and 65 or above) and for treatment type (conservative treatment using immobilisation, or operative treatment by means of internal fixation) to ensure a balanced randomisation into the two groups.

Interventions

After randomisation, patients start participation in the trial as soon as their fracture has been qualified as stable or consolidated, and patients are fit to start active exercises as determined by the clinician; this is either directly after cast removal, or within 3-5 days after successful operative fixation. All patients are instructed by their surgeon to perform hand and wrist rehabilitation exercises 3-5 times per day. An overview of simple exercises for the three main motions in the wrist (palmar/dorsal flexion, pronation/supination and radial/ulnar deviation), is provided on paper. In addition, patients receive a simple diary to complete, with instructions to register their frequency and duration of exercises daily, as well as any physical complaints they may have during exercising.

<u>Intervention group</u>

Patients that are randomized to the game group receive a download link via email, together with instructions on how to download and install the game on their own smartphone or tablet (iPad® or iPhone®, Apple Inc., Cupertino, CA, USA). Patients are instructed to strap one motion sensor to the dorsum of the hand, and to strap a second one to the dorsal side of the proximal forearm (Figure 1). If patients are only in the possession of a compatible smartphone, they will receive the Myo™ gesture control armband (Thalmic Labs Inc. Kitchener, Ontario, Canada). This sensor is worn around the hand, in combination with the patient's own smartphone strapped around the forearm. If patients are only in the possession of a compatible tablet, or if they prefer this option over using their smartphone, they receive two Valedo® sensors (Hocoma AG, Zurich, Switzerland). Two separate

For peer review only - http://bmjopen.bmj.com/site/about/guidelines.xhtml

sensors are used since the tablet cannot be strapped to the arm to be used as a motion sensor. Both types of motion sensors are CE-marked, have been found fit to act as a game controller and are able to monitor wrist range of motion. The sensors are connected to the smartphone via Bluetooth. By using two separate motion sensors placed proximally and distally of the wrist joint, isolated wrist motions are used for game control and patients cannot 'cheat' by moving the shoulder joint or the fingers. All otherwise necessary instructions are embedded within the game, making the game 'self-explanatory'. Intervention group patients will practice with the game for six weeks, after which they return the motion sensor to the hospital and are encouraged to continue exercising independently.

Gameplay

The *ReValidate!* game shows an underwater world in which the patient plays one level consisting of three different mini-games of similar difficulty (Supplementary video). Each mini-game has its own avatar, and is controlled by one specific wrist motion: the 'anglerfish' mini-game is controlled by pronation and supination, the 'shark' mini-game is controlled by palmar and dorsal flexion of the wrist, and the 'penguin' mini-game is controlled by radial an ulnar deviation (Figure 2). The patient plays one level 3-5 times per day. A new level is unlocked each day, so the game remains challenging. Patients are motivated by a daily 'push notification' reminder to exercise, and by optional high score rankings. Bonus points can be gathered by playing consistently 3-5 times per day and by obtaining 'collectibles' found in the different levels. The game application registers frequency and duration of gameplay and records progress in terms of range of motion in degrees.

Because the swimming motions of the fish are comparable to the wrist motions needed to control the mini-games, gameplay through the embedded wrist exercises is intuitive and natural.

The game has been designed specifically for wrist rehabilitation by a company specialized in the development of rehabilitation games. An iterative design process was setup to ensure embedding of the standard exercise protocols. The game has been tested at all stages of development by experts and novices, including trauma surgeons, specialised hand and wrist surgeons, occupational therapists

For peer review only

and physiotherapists, as well as by end-users. Content has been checked for validity and exercise completeness both by user groups of patients and medical experts.[36] Should patients encounter any problems with the game, they are provided contact details of a study coordinator. For any medical questions or concerns, patients are referred back to their treating specialist. Though not preferred, patients receive a referral for additional physiotherapy upon request. They will be motivated by their clinician to continue practicing using the game. The number of physiotherapy visits will be registered prospectively.

Control group

Patients in the control group receive standard care, consisting of home-based unsupervised wrist exercises, and upon request or recommendation by their clinician, a referral for physiotherapy or occupational therapy. This regimen is most reflective of current standard practice.

Outcome measurements

Standard demographic information including age, sex, fracture type according to the AO/OTA (Arbeitsgemeinschaft für Osteosynthesefragen/Orthopaedic Trauma Association) fracture classification,[37] and the type of treatment is obtained directly after inclusion. Follow-up visits take place at two, six, and twelve weeks postoperatively or after cast removal; for operatively or conservatively treated patients, respectively. The patient-rated wrist evaluation (PRWE) questionnaire is completed by patients at all follow-up visits. The active range of motion (ROM) is measured in degrees using an analogue universal goniometer. Range of motion (ROM) and grip strength are measured in both hands, where grip strength is measured in kilograms, using an analogue hand dynamometer (Baseline®, Fabrication Enterprises Inc., White Plains, NY, USA). The maximum value of two measurements with five minutes rest in between is used for analysis. Patients in both study arms complete a questionnaire on their experiences and perceived effectiveness of practice. Pain scores on a visual analogue scale (VAS) are recorded, and patients are

For peer review only - http://bmjopen.bmj.com/site/about/guidelines.xhtml

asked about the frequency, if any, of visits to a physiotherapist or occupational therapist. In addition, partial or complete return to work, or to normal functioning if patients were currently unemployed, is registered. Patients return their completed exercise diary at six weeks of follow-up. In the intervention group, the data on exercise frequency and duration, as well as range of motion, will also be retrieved from the serious game. The frequency reported by patients in this group will be compared to the data from the game application, which stores all exercise data including frequency, duration and the range of motion measured.

Sample size calculation

The primary outcome is the PRWE questionnaire outcome score after 6 weeks of treatment. This questionnaire has been validated previously, and has a minimal clinically important difference (MCID) of 11.5, with 1.5 for the pain subscale, and 10 for the function subscale.[38]

A recent Cochrane review on the effect of physiotherapy after distal radius fractures reports only three studies comparing a regular physiotherapy regimen to an unsupervised home-exercise program.[12] These comparisons are relevant here, since the use of this serious game can be compared to an un supervised home-exercise program. Only one of these three trials reports patient-rated functional outcomes in addition to range of motion or grip strength values.[15] This study looks into the effects of a progressive home-exercise program, compared to the effect of 12 physiotherapy sessions over a 6 week period in patients with an operatively treated distal radius fracture. They found a positive effect in favour of the home-exercise program, with PRWE scores of 18.5 (standard deviation 15.9) for the home-exercise group, compared to 36.1 (SD 13.9) in the physiotherapy group after 6 weeks.[15]

As a result, using the standard deviation of 15.9, for a two-sided t-test in two groups with an alpha of 0.05, a power of 90% (1 – beta = 0.9), an allocation ratio of 1:1, this leads to necessary group sizes of 42 patients per group to detect a clinically relevant difference of 11.5 points at six weeks. With a 10% dropout rate, a total of 92 patients are needed for this trial.

For peer review only - http://bmjopen.bmj.com/site/about/guidelines.xhtml

Data analysis

The primary outcome is the PRWE-score at six weeks. This outcome is represented as the change in outcome scores from T0 to T2. An intention-to-treat analysis is performed, and any missing data is imputed. Data will be analysed using a two-sided t-test or the Mann-Whitney U test, depending on normality of the distribution. A 95% confidence interval not including a PRWE difference of 11.5 points or more will be interpreted as showing no clinically relevant differences in PRWE outcomes between the groups.

The secondary continuous outcomes (PRWE score at 2 and 12 weeks, ROM, grips strength, pain scores), a repeated measures analysis of variance will be performed, where group assignment (game group vs. control group) is the between-subjects factor, and visit (baseline, 2, 6 and 12 weeks) and time of visit (baseline, 2, 6 or 12 weeks follow-up) is the within-subjects factor. An intention-to-treat analysis will be performed, and any missing data will be imputed using multiple imputation. P-values of < 0.05 will be considered statistically significant. Other functional outcomes are analysed as a percentage of the unaffected wrist. Bonferroni correction is applied to correct for multiple tests. Baseline data and secondary outcomes include categorical data. These data will be analysed using the Chi-square test, and outcomes will be presented as the differences in frequencies. P-values of < 0.05 are considered statistically significant. Treatment adherence is measured using a self-reported diary. Frequencies and duration of exercising are continuous variables that will undergo quantitative and qualitative synthesis using a two-sided t-test or a Mann-Whitney U test as appropriate. The number of diaries handed in after the study period are analysed using the Chi-square test, and outcomes will be presented as the difference in frequencies.

For peer review only

For peer review only - http://bmjopen.bmj.com/site/about/guidelines.xhtml

ETHICS AND DISSEMINATION

This study will be conducted according to the principles of the Declaration of Helsinki (64th WMA General Assembly, Fortaleza, Brazil, October 2013) and in accordance with the Medical Research Involving Human Subjects Act (WMO). The dataset will be made available and results of our study will be published unreservedly through academic journals, (international) conferences and popular media.

The medical risks of this trial are considered to be low, since treatment of both the intervention and the control group complies with national guidelines for standard treatment of distal radius fractures. Participation, or the choice to not participate, will not have any effect on any part of patient treatment or on the quality of medical care for patients.

Storing and processing of patient data occurs in compliance with the General Data Protection Act (GDPR), and a data processing agreement has been established between the app manufacturer and the hospital leading the trial. Data gathered by the individual mobile applications has been assessed using a Data Privacy Impact Assessment (DPIA), and approved of by the hospitals' Data Privacy Officer (DPO). All data is stored on a computer server located within the hospital network and can only be accessed by the researchers after logging in with their personal account. The compliance with these rules ensures that privacy risks are minimised.

For peer review only

DISCUSSION

There is a rapid increase in the use of technological applications for healthcare support and patient self-management.[39-41] Clinical research on the effectiveness of these innovative applications is still scarce, however.[31] 'Wearables' show potential as monitoring devices, since they are non-obtrusive and can monitor patients over longer periods of time.[42] In addition, serious games have shown their own potential in education, improving both motivation and learning outcomes,[43,44] and have also shown to be promising tools to increase self-efficacy; an important factor contributing to treatment adherence and physiotherapy outcomes.[26,28]

Readily available computer games using body motion for control, for example the Nintendo® Wii™, have already been researched in clinical settings as treatment support tools.[41] Though, the issue with off-the-shelf games is that they are not designed as a medical device, hence lack proper validation for medical use. They may increase activity and motivation in a rehabilitation process, yet have been designed for entertainment purposes.[36] Furthermore, these games usually require larger consoles that limit the patient in performing the exercises anywhere they want.

To the authors' knowledge, this study is the first study focusing on a validated, wearable-controlled serious game designed to act as a medical support tool for wrist rehabilitation. This game and similar interventions may decrease the ever-growing burden wrist injuries pose to patients and to society, and can make validated wrist exercise therapy easily accessible for anyone. This study will contribute to the advancements of serious games for traumatic and non-traumatic wrist and other injuries, and may pave the way for future development and validation of wearable-controlled rehabilitation games.

For peer review only - http://bmjopen.bmj.com/site/about/guidelines.xhtml

ACKNOWLEDGEMENTS

We acknowledge all physiotherapists and hand therapists that have collaborated during development of the game. We especially thank A.J. Videler of the Hand- and Wrist Centre, Amsterdam, and C.J.M. Nooij of the department of Rehabilitation, Amsterdam UMC, location Academic Medical Centre. We thank Motek Medical B.V. and Virtual Play for the collaborative development of the game. Furthermore, we thank all supporting physicians, specialised nurses and other nurses, and outpatient clinic staff from all collaborating hospitals that made the realization of this trial possible.

FOOTNOTES

Author contributions:

All authors have contributed to the design of this trial protocol. MPS and JCG initiated the project. The protocol was drafted by HAWM, and revised by MCO, MG, MPS, SVD and JCG. SVD provided statistical and methodological support. All authors have read and approved the final manuscript. The ReValidate! collaborative study group consists of local investigators who are responsible for trial execution, patient inclusion and data collection. They have all read and approved the final manuscript.

Conflict of interest statement:

The authors have no conflicts of interest to declare.

Funding:

The ReValidate! project received funding by the Growing Games Program (Dutch Game Garden, iMMovator, Dutch Games Association and the Economic Board Utrecht), grant number CB00018, by a grant from the CZ Fund, the Netherlands. The sponsor had no role of any sort in the study design.

For peer review only - http://bmjopen.bmj.com/site/about/guidelines.xhtml

Patient consent:

Obtained

Ethics approval:

This study has been evaluated and approved by the Medical Ethical Review Board at the Academic Medical Centre, Amsterdam, the Netherlands.

Collaborators:

Prof. P. Kloen, MD PhD, Amsterdam UMC, location Academic Medical Centre, Amsterdam, the Netherlands;

H.W. Bolhuis, MD PhD, Gelre ziekenhuizen, Apeldoorn, the Netherlands;

S.R. de Wild, MD, Gelre Ziekenhuizen, Apeldoorn, the Netherlands;

T. Herklots, MD, Gelre Ziekenhuizen, Apeldoorn, the Netherlands;

J. Baaij, MD MSc, Gelre Ziekenhuizen, Apeldoorn, the Netherlands;

J. Winkelhagen, MD, Dijklanderziekenhuis, Hoorn, the Netherlands;

R. Buijsman, MD, Amsterdam UMC, location Academic Medical Centre, Amsterdam, the Netherlands;

K.T. van Hamersveld, MD, Dijklanderziekenhuis, Hoorn, the Netherlands;

B.A. van Dijkman, MD PhD, Flevoziekenhuis, Almere, the Netherlands;

M. Jansen, MD, Amsterdam UMC, location Academic Medical Centre, Amsterdam, the Netherlands;

E.Z. Barsom, MD, Amsterdam UMC, location Academic Medical Centre, Amsterdam, the Netherlands;

S.L. van der Storm, MD, Amsterdam UMC, location Academic Medical Centre, Amsterdam, the Netherlands;

N. van Oorschot, MD, Amsterdam UMC, location Academic Medical Centre, Amsterdam, the Netherlands;

M.J. van der Pols, Amsterdam UMC, location Academic Medical Centre, Amsterdam, the Netherlands.

For peer review only - http://bmjopen.bmj.com/site/about/guidelines.xhtml

For peer review only

REFERENCES

1. Court-Brown CM, Caesar B. Epidemiology of adult fractures: A review. *Injury* 2006;37(8):691-7. doi: 10.1016/j.injury.2006.04.130 [published Online First: 2006/07/04]

2. Kilgore ML, Morrisey MA, Becker DJ, et al. Health care expenditures associated with skeletal fractures among Medicare beneficiaries, 1999-2005. *J Bone Miner Res* 2009;24(12):2050-5. doi: 10.1359/jbmr.090523 [published Online First: 2009/05/21]

3. Nellans KW, Kowalski E, Chung KC. The epidemiology of distal radius fractures. *Hand Clin* 2012;28(2):113-25. doi: 10.1016/j.hcl.2012.02.001 [published Online First: 2012/05/05]

4. Chung KC, Spilson SV. The frequency and epidemiology of hand and forearm fractures in the United States. *J Hand Surg Am* 2001;26(5):908-15. doi: 10.1053/jhsu.2001.26322 [published Online First: 2001/09/19]

5. Brogren E, Petranek M, Atroshi I. Incidence and characteristics of distal radius fractures in a southern Swedish region. *BMC Musculoskelet Disord* 2007;8:48. doi: 10.1186/1471-2474-8-48 [published Online First: 2007/06/02]

6. MacIntyre NJ, Dewan N. Epidemiology of distal radius fractures and factors predicting risk and prognosis. *J Hand Ther* 2016;29(2):136-45. doi: 10.1016/j.jht.2016.03.003 [published Online First: 2016/06/07]

7. Stirling ERB, Johnson NA, Dias JJ. Epidemiology of distal radius fractures in a geographically defined adult population. *Journal of Hand Surgery (European Volume)* 2018;43(9):974-82. doi: 10.1177/1753193418786378

8. Angermann P, Lohmann M. Injuries to the hand and wrist. A study of 50,272 injuries. *J Hand Surg Br* 1993;18(5):642-4. doi: 10.1016/0266-7681(93)90024-a [published Online First: 1993/10/01]

9. de Putter CE, Selles RW, Polinder S, et al. Economic impact of hand and wrist injuries: health-care costs and productivity costs in a population-based study. *J Bone Joint Surg Am* 2012;94(9):e56. doi: 10.2106/JBJS.K.00561 [published Online First: 2012/05/04]

For peer review only

For peer review only - http://bmjopen.bmj.com/site/about/guidelines.xhtml

10. Larsen CF, Mulder S, Johansen AMT, et al. The epidemiology of hand injuries in the Netherlands and Denmark. *Eur J Epidemiol* 2004;19(4):323-27.

11. Fricker R, Jupiter J, Kastelec M. AO Surgery reference: Distal forearm [Web page]. Davos, Switzerland: AO Foundation; 2017 [Available from: https://www2.aofoundation.org/wps/portal/surgery?showPage=diagnosis&bone=Radius&segment=Distal accessed Jun 4 2017.

12. Handoll HHG, Elliott J. Rehabilitation for distal radial fractures in adults (Cochrane review) [with consumer summary]. *Cochrane Database of Systematic Reviews 2005;Issue 9* 2015

13. Bruder AM, Shields N, Dodd KJ, et al. A progressive exercise and structured advice program does not improve activity more than structured advice alone following a distal radial fracture: a multi-centre, randomised trial. *Journal of physiotherapy* 2016;62(3):145-52

14. Gutierrez-Espinoza H, Rubio-Oyarzun D, Olguin-Huerta C, et al. Supervised physical therapy vs home exercise program for patients with distal radius fracture: A single-blind randomized clinical study. *Journal of Hand Therapy* 2017;07

15. Krischak G, Krasteva A, Schneider F, et al. Physiotherapy after volar plating of wrist fractures is effective using a home exercise program. *Archives of physical medicine and rehabilitation* 2009; 90(4).

16. Brehmer JL, Husband JB. Accelerated rehabilitation compared with a standard protocol after distal radial fractures treated with volar open reduction and internal fixation: A prospective, randomized, controlled study. *Journal of Bone and Joint Surgery - American Volume* 2014; 96(19)

17. Brehmer JL, Husband JB. Accelerated rehabilitation compared with a standard protocol after distal radial fractures treated with volar open reduction and internal fixation: A prospective, randomized, controlled study. *Journal of Bone and Joint Surgery - American Volume* 2014;96(19):1621-30

For peer review only - http://bmjopen.bmj.com/site/about/guidelines.xhtml

18. Lozano-Calderón S, Souer S, Mudgal C, et al. Wrist mobilization following volar plate fixation of fractures of the distal part of the radius. *The Journal of bone and joint surgery American volume* 2008; 90(6)

19. Kay S, McMahon M, Stiller K. An advice and exercise program has some benefits over natural recovery after distal radius fracture: a randomised trial. *The Australian journal of physiotherapy* 2008; 54(4)

20. American Academy of Orthopaedic Surgeons. The treatment of distal radius fractures - Guideline and evidence report. Recommendation 21-23. Rosemont, IL: AAOS, 2009:84-92.

21. Deutschen Gesellschaft für Unfallchirurgie e.V. (DGU). Distale Radiusfraktur - Leitlinie Unfallchirurgie. Physiotherapie. Göttingen: DGU, 2014:32-33.

22. Nederlandse Vereniging voor Heelkunde. Richtlijn Distale Radiusfracturen: diagnostiek en behandeling., 2010.

23. Heelkunde; NVv. Richtlijn Distale Radiusfracturen: diagnostiek en behandeling. Utrecht, 2010.

24. Sluijs EM, Kok GJ, van der Zee J. Correlates of exercise compliance in physical therapy. *Phys Ther* 1993;73(11):771-82; discussion 83-6. doi: 10.1093/ptj/73.11.771 [published Online First: 1993/11/01]

25. Martin LR, Williams SL, Haskard KB, et al. The challenge of patient adherence. *Ther Clin Risk Manag* 2005;1(3):189-99. [published Online First: 2008/03/25]

26. Picha KJ, Howell DM. A model to increase rehabilitation adherence to home exercise programmes in patients with varying levels of self-efficacy. *Musculoskeletal Care* 2018;16(1):233-37. doi: 10.1002/msc.1194 [published Online First: 2017/04/13]

27. Lyngcoln A, Taylor N, Pizzari T, et al. The relationship between adherence to hand therapy and short-term outcome after distal radius fracture. *J Hand Ther* 2005;18(1):2-8; quiz 9. doi: 10.1197/j.jht.2004.10.008 [published Online First: 2005/01/28]

For peer review only - http://bmjopen.bmj.com/site/about/guidelines.xhtml

28. Bjork M, Niklasson J, Westerdahl E, et al. Self-efficacy corresponds to wrist function after combined plating of distal radius fractures. *J Hand Ther* 2020 doi: 10.1016/j.jht.2020.01.001 [published Online First: 2020/02/24]

29. Burke JW, McNeill MDJ, Charles DK, et al. Optimising engagement for stroke rehabilitation using serious games. *Visual Comput* 2009;25(12):1085-99. doi: 10.1007/s00371-009-0387-4

30. Laver KE, George S, Thomas S, et al. Virtual reality for stroke rehabilitation. *The Cochrane database of systematic reviews* 2015;2:CD008349. doi: 10.1002/14651858.CD008349.pub3

31. Meijer HA, Graafland M, Goslings JC, et al. Systematic Review on the Effects of Serious Games and Wearable Technology Used in Rehabilitation of Patients With Traumatic Bone and Soft Tissue Injuries. *Arch Phys Med Rehabil* 2018;99(9):1890-99. doi: 10.1016/j.apmr.2017.10.018 [published Online First: 2017/11/16]

32. Bergeron BP. Developing Serious Games. 1st ed. Hingham, MA: Charles River Media 2006.

33. Brichetto G, Spallarossa P, de Carvalho ML, et al. The effect of Nintendo(R) Wii(R) on balance in people with multiple sclerosis: a pilot randomized control study. *Multiple sclerosis (Houndmills, Basingstoke, England)* 2013;19(9):1219-21. doi: 10.1177/1352458512472747 [published Online First: 2013/01/17]

34. Butler DP, Willett K. Wii-habilitation: is there a role in trauma? *Injury* 2010;41(9):883-5. doi: 10.1016/j.injury.2010.03.024 [published Online First: 2010/04/20]

35. Morone G, Tramontano M, Iosa M, et al. The efficacy of balance training with video game-based therapy in subacute stroke patients: a randomized controlled trial. *BioMed research international* 2014;2014:580861. doi: 10.1155/2014/580861 [published Online First: 2014/05/31]

36. Meijer HAW, Graafland M, Obdeijn MC, et al. Face Validity and Content Validity of a Game for Distal Radius Fracture Rehabilitation. *J Wrist Surg* 2019;8(5):388-94. doi: 10.1055/s-0039-1688948 [published Online First: 2019/10/04]

For peer review only

37. Meinberg EG, Agel J, Roberts CS, et al. Fracture and Dislocation Classification Compendium-2018. *J Orthop Trauma* 2018;32 Suppl 1:S1-S170. doi: 10.1097/BOT.0000000000001063 [published Online First: 2017/12/20]

38. Walenkamp MM, de Muinck Keizer RJ, Goslings JC, et al. The Minimum Clinically Important Difference of the Patient-rated Wrist Evaluation Score for Patients With Distal Radius Fractures. *Clin Orthop Relat Res* 2015;473(10):3235-41. doi: 10.1007/s11999-015-4376-9

39. Arrebola LS, Yi LC, De Oliveira VGC. The use of video games combined with conventional physical therapy in children with upper limb fractures: An exploratory study. *Journal of Pediatric Rehabilitation Medicine* 2019;12(1):65-70

40. Bruder AM, McClelland JA, Shields N, et al. Validity and reliability of an activity monitor to quantify arm movements and activity in adults following distal radius fracture. *Disability & Rehabilitation* 2018;40(11):1318-25. doi: 10.1080/09638288.2017.1288764

41. Laver KE, Lange B, George S, et al. Virtual reality for stroke rehabilitation. *Cochrane Database Syst Rev* 2017;11:CD008349. doi: 10.1002/14651858.CD008349.pub4 [published Online First: 2017/11/21]

42. Bonato P. Wearable sensors/systems and their impact on biomedical engineering. *IEEE engineering in medicine and biology magazine : the quarterly magazine of the Engineering in Medicine & Biology Society* 2003;22(3):18-20.

43. Graafland M, Bemelman WA, Schijven MP. Appraisal of face and content validity of a serious game improving situational awareness in surgical training. *Journal of laparoendoscopic & advanced surgical techniques Part A* 2015;25(1):43-9. doi: 10.1089/lap.2014.0043

44. Graafland M, Vollebergh MF, Lagarde SM, et al. A serious game can be a valid method to train clinical decision-making in surgery. *World journal of surgery* 2014;38(12):3056-62. doi: 10.1007/s00268-014-2743-4

For peer review only - http://bmjopen.bmj.com/site/about/guidelines.xhtml

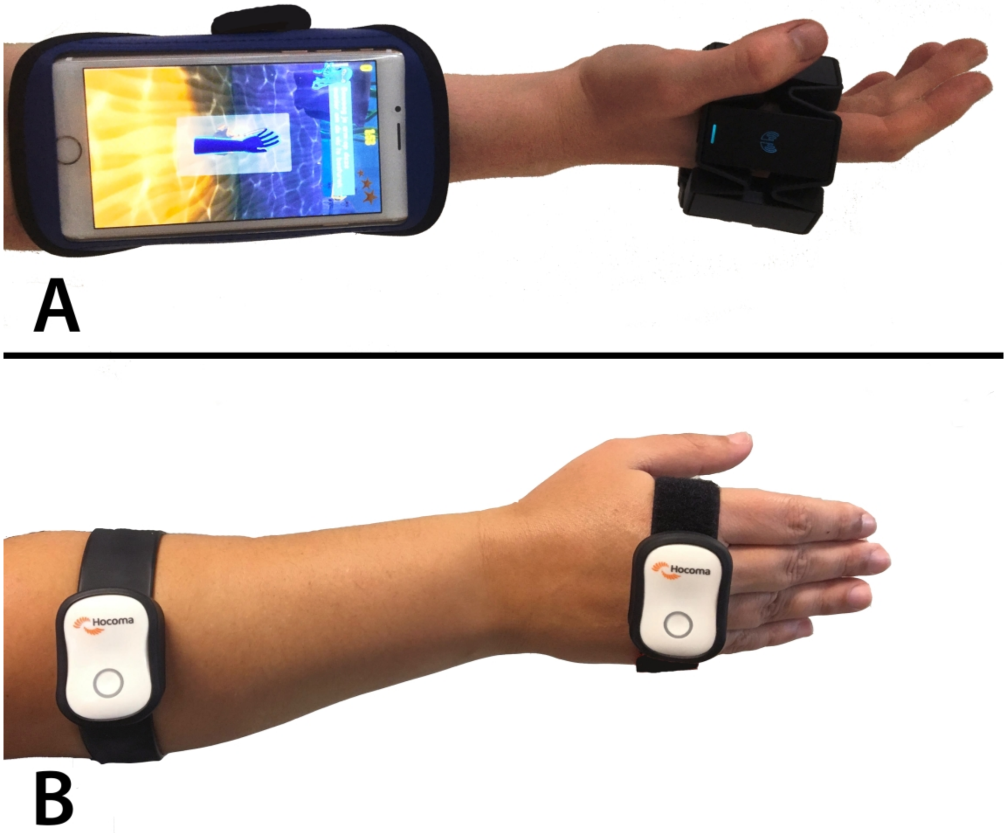

Figure 1: Sensor placement. A: iPhone® and Myo™; B: two Valedo™ sensors.

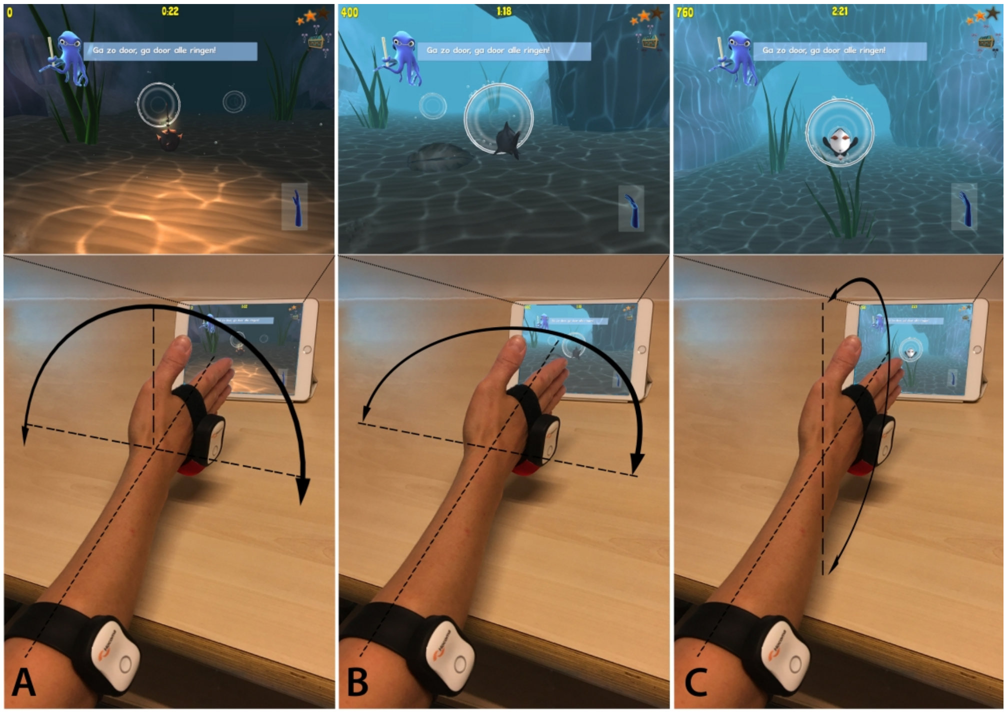

Figure 2: Different avatars are used in the game. A: the anglerfish is controlled by pronation and supination; B: the shark is controlled by palmar and dorsal flexion; C: the penguin is controlled by radial and ulnar deviation.

STANDARD PROTOCOL ITEMS: RECOMMENDATIONS FOR INTERVENTIONAL TRIALS

SPIRIT 2013 Checklist: Recommended items to address in a clinical trial protocol and related documents*

Section/item	Item No	Description	Addressed on page number
**Administrative information**			
Title	1	Descriptive title identifying the study design, population, interventions, and, if applicable, trial acronym	1
Trial registration	2a	Trial identifier and registry name. If not yet registered, name of intended registry	3
	2b	All items from the World Health Organization Trial Registration Data Set	3
Protocol version	3	Date and version identifier	3
Funding	4	Sources and types of financial, material, and other support	14
Roles and responsibilities	5a	Names, affiliations, and roles of protocol contributors	1, 15
	5b	Name and contact information for the trial sponsor	1, 14
	5c	Role of study sponsor and funders, if any, in study design; collection, management, analysis, and interpretation of data; writing of the report; and the decision to submit the report for publication, including whether they will have ultimate authority over any of these activities	14
	5d	Composition, roles, and responsibilities of the coordinating centre, steering committee, endpoint adjudication committee, data management team, and other individuals or groups overseeing the trial, if applicable (see Item 21a for data monitoring committee)	14

For peer review only

Introduction

Background and rationale	6a	Description of research question and justification for undertaking the trial, including summary of relevant studies (published and unpublished) examining benefits and harms for each intervention	4-5
	6b	Explanation for choice of comparators	4
Objectives	7	Specific objectives or hypotheses	5
Trial design	8	Description of trial design including type of trial (eg, parallel group, crossover, factorial, single group), allocation ratio, and framework (eg, superiority, equivalence, noninferiority, exploratory)	5, 6

Methods: Participants, interventions, and outcomes

Study setting	9	Description of study settings (eg, community clinic, academic hospital) and list of countries where data will be collected. Reference to where list of study sites can be obtained	6
Eligibility criteria	10	Inclusion and exclusion criteria for participants. If applicable, eligibility criteria for study centres and individuals who will perform the interventions (eg, surgeons, psychotherapists)	6
Interventions	11a	Interventions for each group with sufficient detail to allow replication, including how and when they will be administered	6-8
	11b	Criteria for discontinuing or modifying allocated interventions for a given trial participant (eg, drug dose change in response to harms, participant request, or improving/worsening disease)	9
	11c	Strategies to improve adherence to intervention protocols, and any procedures for monitoring adherence (eg, drug tablet return, laboratory tests)	8
	11d	Relevant concomitant care and interventions that are permitted or prohibited during the trial	10
Outcomes	12	Primary, secondary, and other outcomes, including the specific measurement variable (eg, systolic blood pressure), analysis metric (eg, change from baseline, final value, time to event), method of aggregation (eg, median, proportion), and time point for each outcome. Explanation of the clinical relevance of chosen efficacy and harm outcomes is strongly recommended	9, 11
Participant timeline	13	Time schedule of enrolment, interventions (including any run-ins and washouts), assessments, and visits for participants. A schematic diagram is highly recommended (see Figure)	7

Sample size	14	Estimated number of participants needed to achieve study objectives and how it was determined, including clinical and statistical assumptions supporting any sample size calculations	10
Recruitment	15	Strategies for achieving adequate participant enrolment to reach target sample size	6, otherwise not described

Methods: Assignment of interventions (for controlled trials)

Allocation:

Sequence generation	16a	Method of generating the allocation sequence (eg, computer-generated random numbers), and list of any factors for stratification. To reduce predictability of a random sequence, details of any planned restriction (eg, blocking) should be provided in a separate document that is unavailable to those who enrol participants or assign interventions	7
Allocation concealment mechanism	16b	Mechanism of implementing the allocation sequence (eg, central telephone; sequentially numbered, opaque, sealed envelopes), describing any steps to conceal the sequence until interventions are assigned	7
Implementation	16c	Who will generate the allocation sequence, who will enrol participants, and who will assign participants to interventions	7
Blinding (masking)	17a	Who will be blinded after assignment to interventions (eg, trial participants, care providers, outcome assessors, data analysts), and how	7
	17b	If blinded, circumstances under which unblinding is permissible, and procedure for revealing a participant's allocated intervention during the trial	not described

Methods: Data collection, management, and analysis

Data collection methods	18a	Plans for assessment and collection of outcome, baseline, and other trial data, including any related processes to promote data quality (eg, duplicate measurements, training of assessors) and a description of study instruments (eg, questionnaires, laboratory tests) along with their reliability and validity, if known. Reference to where data collection forms can be found, if not in the protocol	11
	18b	Plans to promote participant retention and complete follow-up, including list of any outcome data to be collected for participants who discontinue or deviate from intervention protocols	8, not otherwise described

Data management	19	Plans for data entry, coding, security, and storage, including any related processes to promote data quality (eg, double data entry; range checks for data values). Reference to where details of data management procedures can be found, if not in the protocol	11
Statistical methods	20a	Statistical methods for analysing primary and secondary outcomes. Reference to where other details of the statistical analysis plan can be found, if not in the protocol	11
	20b	Methods for any additional analyses (eg, subgroup and adjusted analyses)	11
	20c	Definition of analysis population relating to protocol non-adherence (eg, as randomised analysis), and any statistical methods to handle missing data (eg, multiple imputation)	11
**Methods: Monitoring**			
Data monitoring	21a	Composition of data monitoring committee (DMC); summary of its role and reporting structure; statement of whether it is independent from the sponsor and competing interests; and reference to where further details about its charter can be found, if not in the protocol. Alternatively, an explanation of why a DMC is not needed	not specified
	21b	Description of any interim analyses and stopping guidelines, including who will have access to these interim results and make the final decision to terminate the trial	no interim analysis performed
Harms	22	Plans for collecting, assessing, reporting, and managing solicited and spontaneously reported adverse events and other unintended effects of trial interventions or trial conduct	not specified
Auditing	23	Frequency and procedures for auditing trial conduct, if any, and whether the process will be independent from investigators and the sponsor	not specified
**Ethics and dissemination**			
Research ethics approval	24	Plans for seeking research ethics committee/institutional review board (REC/IRB) approval	12
Protocol amendments	25	Plans for communicating important protocol modifications (eg, changes to eligibility criteria, outcomes, analyses) to relevant parties (eg, investigators, REC/IRBs, trial participants, trial registries, journals, regulators)	not specified

Consent or assent	26a	Who will obtain informed consent or assent from potential trial participants or authorised surrogates, and how (see Item 32)	12, not otherwise specified
	26b	Additional consent provisions for collection and use of participant data and biological specimens in ancillary studies, if applicable	not specified
Confidentiality	27	How personal information about potential and enrolled participants will be collected, shared, and maintained in order to protect confidentiality before, during, and after the trial	not specified
Declaration of interests	28	Financial and other competing interests for principal investigators for the overall trial and each study site	14
Access to data	29	Statement of who will have access to the final trial dataset, and disclosure of contractual agreements that limit such access for investigators	12
Ancillary and post-trial care	30	Provisions, if any, for ancillary and post-trial care, and for compensation to those who suffer harm from trial participation	not specified
Dissemination policy	31a	Plans for investigators and sponsor to communicate trial results to participants, healthcare professionals, the public, and other relevant groups (eg, via publication, reporting in results databases, or other data sharing arrangements), including any publication restrictions	12
	31b	Authorship eligibility guidelines and any intended use of professional writers	14
	31c	Plans, if any, for granting public access to the full protocol, participant-level dataset, and statistical code	12
**Appendices**			
Informed consent materials	32	Model consent form and other related documentation given to participants and authorised surrogates	not described
Biological specimens	33	Plans for collection, laboratory evaluation, and storage of biological specimens for genetic or molecular analysis in the current trial and for future use in ancillary studies, if applicable	not applicable, not described

*It is strongly recommended that this checklist be read in conjunction with the SPIRIT 2013 Explanation & Elaboration for important clarification on the items. Amendments to the protocol should be tracked and dated. The SPIRIT checklist is copyrighted by the SPIRIT Group under the Creative Commons "Attribution-NonCommercial-NoDerivs 3.0 Unported" license.

For peer review only - http://bmjopen.bmj.com/site/about/guidelines.xhtml

BMJ Open

A serious game versus standard care for rehabilitation after distal radius fractures: a protocol for a multicentre randomised controlled trial

Journal:	*BMJ Open*
Manuscript ID	bmjopen-2020-042629.R1
Article Type:	Protocol
Date Submitted by the Author:	20-Nov-2020
Complete List of Authors:	Meijer, Henriëtte; Amsterdam UMC Locatie AMC, Department of Surgery, Amsterdam Movement Sciences<br>Graafland, Maurits; Amsterdam UMC Locatie AMC, Department of Surgery<br>Obdeijn, Miryam; Amsterdam UMC Locatie AMC, Department of Plastic, Reconstructive and Hand Surgery<br>van Dieren, Susan; Amsterdam UMC Locatie AMC, Department of Surgery<br>Goslings, J. Carel; Onze Lieve Vrouwe Gasthuis, Department of Trauma Surgery<br>Schijven, Marlies; Amsterdam UMC Locatie AMC, Department of Surgery, Amsterdam Gastroenterology and Metabolism<br>ReValidate!, Collaborative research group; Amsterdam UMC Locatie AMC, Department of Surgery
**Primary Subject Heading**:	Rehabilitation medicine
Secondary Subject Heading:	Health informatics, Surgery
Keywords:	Hand & wrist < ORTHOPAEDIC & TRAUMA SURGERY, REHABILITATION MEDICINE, Telemedicine < BIOTECHNOLOGY & BIOINFORMATICS, BIOTECHNOLOGY & BIOINFORMATICS, Information technology < BIOTECHNOLOGY & BIOINFORMATICS, Orthopaedic & trauma surgery < SURGERY

Note: The following files were submitted by the author for peer review, but cannot be converted to PDF. You must view these files (e.g. movies) online.

ReValidate! - Wrist fracture rehabilitation.wmv

SCHOLARONE™ Manuscripts

I, the Submitting Author has the right to grant and does grant on behalf of all authors of the Work (as defined in the below author licence), an exclusive licence and/or a non-exclusive licence for contributions from authors who are: i) UK Crown employees; ii) where BMJ has agreed a CC-BY licence shall apply, and/or iii) in accordance with the terms applicable for US Federal Government officers or employees acting as part of their official duties; on a worldwide, perpetual, irrevocable, royalty-free basis to BMJ Publishing Group Ltd ("BMJ") its licensees and where the relevant Journal is co-owned by BMJ to the co-owners of the Journal, to publish the Work in this journal and any other BMJ products and to exploit all rights, as set out in our licence.

The Submitting Author accepts and understands that any supply made under these terms is made by BMJ to the Submitting Author unless you are acting as an employee on behalf of your employer or a postgraduate student of an affiliated institution which is paying any applicable article publishing charge ("APC") for Open Access articles. Where the Submitting Author wishes to make the Work available on an Open Access basis (and intends to pay the relevant APC), the terms of reuse of such Open Access shall be governed by a Creative Commons licence – details of these licences and which Creative Commons licence will apply to this Work are set out in our licence referred to above.

Other than as permitted in any relevant BMJ Author's Self Archiving Policies, I confirm this Work has not been accepted for publication elsewhere, is not being considered for publication elsewhere and does not duplicate material already published. I confirm all authors consent to publication of this Work and authorise the granting of this licence.

For peer review only

A serious game versus standard care for rehabilitation after distal radius fractures: a protocol for a multicentre randomised controlled trial

H.A.W. Meijer, MD MSc[1], M. Graafland, MD PhD[2], M.C. Obdeijn, MD PhD[3], S. van Dieren, PhD[4], J.C. Goslings, MD PhD[5], M.P. Schijven, MD PhD MHSc[6], on behalf of the ReValidate!-collaborative study group

1: Department of Surgery, Amsterdam Movement Sciences, Amsterdam UMC, University of Amsterdam, Meibergdreef 9, P.O. Box 22660, 1100 DD Amsterdam, the Netherlands. Email: h.a.meijer@amsterdamumc.nl
2: Department of Surgery, Amsterdam UMC, University of Amsterdam, Meibergdreef 9, P.O. Box 22660, 1100 DD Amsterdam, the Netherlands. Email: m.graafland@amsterdamumc.nl
3: Department of Plastic, Reconstructive and Hand Surgery, Amsterdam UMC, University of Amsterdam, Meibergdreef 9, P.O. Box 22660, 1100 DD Amsterdam, the Netherlands. Email: m.c.obdeijn@amsterdamumc.nl
4: Department of Surgery, Amsterdam UMC, University of Amsterdam, Meibergdreef 9, P.O. Box 22660, 1100 DD Amsterdam, the Netherlands. Email: s.vandieren@amsterdamumc.nl
5: Department of Trauma Surgery, Onze Lieve Vrouwe Gasthuis, Jan Tooropstraat 164, P.O. Box 9243, 1006 AE Amsterdam, the Netherlands. Email: j.c.gosling@olvg.nl
6: Department of Surgery, Amsterdam Gastroenterology and Metabolism, Amsterdam UMC, University of Amsterdam, Meibergdreef 9, P.O. Box 22660, 1100 DD Amsterdam, the Netherlands. Email: m.p.schijven@amsterdamumc.nl

Corresponding author:

Prof. dr. M.P. Schijven, Department of Surgery, Amsterdam Gastroenterology and Metabolism, Amsterdam UMC, University of Amsterdam, Meibergdreef 9, P.O. Box 22660, 1100 DD Amsterdam, the Netherlands. Phone: +31 20 5664207. Email: m.p.schijven@amsterdamumc.nl

Word count:

Abstract: 293 words

Manuscript: 3666 words

For peer review only - http://bmjopen.bmj.com/site/about/guidelines.xhtml

ABSTRACT

Introduction

Distal radius fractures are amongst the most prevalent traumatic injuries worldwide. These injuries are associated with high healthcare-related and socio-economic costs, mainly resulting from loss of productivity. To optimise recovery and return to work, wrist exercises are recommended. However, adherence to standard exercise regimens is low. Serious games provide a treatment platform for standardised postoperative care, uniting meaningful recovery with entertainment. Also, mobile serious games, for example smartphone or tablet applications, are able to send practice reminders believed to improve self-efficacy.

Methods and analysis

To test the effectiveness of a mobile serious game for distal radius fracture rehabilitation compared to standard care, a multicentre, randomised controlled clinical trial was designed. Primary outcome will be the Patient-Rated Wrist Evaluation (PRWE) score after six weeks of treatment. Secondary outcomes are range of motion, grip strength, pain scores and self-reported treatment adherence after two, six and twelve weeks of treatment.

Adult patients with any type of closed distal radius fracture are included directly after non-operative or operative fracture treatment. Patients are recruited in the outpatient clinics of four teaching hospitals. The intended sample size is 92 patients, based on the minimal clinically important difference of the PRWE score at six weeks, using a superiority model.

Patients are randomised between using the wearable-controlled mobile serious game *ReValidate!* (intervention group), or standard care consisting of unsupervised exercises and a referral for physiotherapy or exercise therapy upon request or recommendation by the treating clinician (control group).

Ethics and dissemination

For peer review only

The protocol has been approved by the Medical Ethical Review Board of the Amsterdam University Medical Centres, location Academic Medical Centre in Amsterdam, the Netherlands. Results will be made available to involved healthcare providers, funders, and to the general public including patients via peer-reviewed academic journals and international conferences.

ARTICLE SUMMARY

Strengths and limitations of this study

- This study is a randomised controlled trial comparing a wearable-controlled mobile serious game for wrist rehabilitation to standard care, the first in its kind to the authors' knowledge.
- The study is sufficiently powered to show a clinically important difference in the outcome measurements of the primary endpoint.
- By measuring treatment adherence both subjectively and objectively, a reliable comparison between patient-reported and objectively monitored treatment adherence can be made.
- The control group receives an accurate representation of the current standard of care.
- The analysing statistician will be blinded to randomisation group.

Keywords:

Distal radius fracture; Rehabilitation; Physical therapy; e-Health; Serious game

Trial registration number: Dutch Trial Registry (NTR), NL6140, 12-04-2017, protocol version 2.

For peer review only

INTRODUCTION

Distal radius fractures are amongst the most frequently occurring traumatic injuries worldwide, occurring in 18% of patients that present to the Emergency Department after having sustained any fracture.[1-3] Up to 25% of fractures occurring in paediatric patients and 18% of fractures in the elderly population are distal radius fractures,[1,4] with an incidence of 30 and 25 per 10,000 person-years for the under 18 and over 65 years old age groups, respectively.[5] In the age group over 65 years old, the incidence in women is five times higher than that in men.[4,6] As the general population ages, the incidence of these injuries is increasing.[1,6-9] Not only are these injuries a burden to patients affected, they also present a significant socio-economic burden to society. Hand and wrist injuries are expensive mainly due to the loss of productivity. Distal radius fractures form the largest and most expensive group within this population.[10-13]

Distal radius fractures can be treated either operatively or conservatively.[14] In order to speed up and enhance recovery of mobility and function after initial fracture treatment, patients are often referred for physiotherapy or hand therapy. Yet, currently available scientific literature remains inconclusive on whether supervised physiotherapy or unsupervised exercise programmes should be advocated. [15-18] There is consensus that starting exercises early is preferred over starting later,[19,20] and that performing any type of exercise is most likely more effective than performing no exercises.[21]

Similarly, surgical guidelines from various countries state that exercises are most likely beneficial.[22-25] These guidelines mostly leave it up to the clinician's preferences whether or not to refer a patient to a physiotherapist, or recommend a referral when patient suffer severe pain or complaints of oedema.[15,22-25] This may lead to arbitrary rehabilitation regimens differing per region, hospital, and even per individual healthcare provider. It is currently unknown what percentage of patients follow-up to physiotherapy referrals. When patients are referred for physiotherapy and follow up to this referral, it has been found that only 19-35% of prescribed exercise regimens are executed completely and correctly.[26] Treatment adherence is influenced by practical constraints such as

For peer review only - http://bmjopen.bmj.com/site/about/guidelines.xhtml

time, costs and travel distances.[27] Patients with a low self-efficacy will also perceive more barriers to treatment adherence in a home-based exercise programme.[28] Self-efficacy is defined as the belief one can be successful when performing a certain task, and is an important contributor to physiotherapy outcomes.[29,30]

In physiotherapy and rehabilitation, so-called ‘serious games’ have been gaining attention for their presumed effects on motivation and functional outcomes.[31-33] A serious game is defined as any kind of interactive computer application that incorporates gamification principles and serves an educational purpose, or aims to achieve a predefined goal.[34] Most studies evaluating the effect of serious games in physiotherapy, use consumer-based game consoles with an external hardware component, for example the Nintendo Wii™.[35-37] These types of games enable patients to perform rehabilitation exercises. As a downside, such games require a game-console and a monitor to function, limiting patients in their ability to exercise anywhere they want. A systematic review evaluating the use of serious games in rehabilitation of traumatic injuries described only ‘off-the-shelf’ games. No ‘wearable-controlled’ games could be identified.[32] Wearable sensors combined with easily accessible gaming platforms such as smartphones or tablets allow the user to exercise anytime and anywhere they want, and can decrease barriers to treatment adherence. To the best of our knowledge, there is no previous research on a home-based wearable-controlled serious game for traumatic injuries.

This multicentre, randomised controlled clinical trial aims to evaluate the effects on functional outcomes of a wearable-controlled serious game played on a smartphone or tablet, that is developed specifically for wrist rehabilitation. This is compared with the current standard treatment following distal radius fracture. This study hypothesizes that the use of the game may lead to a higher treatment adherence by improving motivation and adding entertainment to exercises, hereby increasing self-efficacy. It is therefore hypothesized that patients recovering with use of the game will show a greater improvement in patient-rated functional outcomes compared to standard treatment. Secondary, this study aims to compare active range of motion, grip strength, treatment

For peer review only - http://bmjopen.bmj.com/site/about/guidelines.xhtml

adherence, pain scores and to measure the effects on return to work between the two treatment strategies.

For peer review only

For peer review only - http://bmjopen.bmj.com/site/about/guidelines.xhtml

METHODS AND ANALYSIS

Study design

The study is a two-arm, parallel-group, multicentre randomised controlled superiority trial, designed to evaluate the effectiveness of a wearable-controlled serious game played on a smartphone or a tablet for distal radius fracture rehabilitation. Patients are randomised in a 1:1 ratio.

Participants and setting

Consecutive patients aged 18 years or older, with any type of conservatively or operatively treated closed distal radius fracture, are eligible to participate in the study. Table 1 provides a complete overview of inclusion and exclusion criteria. Patients are recruited from four different teaching hospitals in the country, of which one academic centre. Patients are recruited at the outpatient clinics, where clinicians will be approached for permission to contact their patients about the study. Before starting trial participation, written informed consent will be obtained from all participants by the research staff.

Patients with a medical history of loss of function due to injury or illness in either hand or wrist, polytraumatised patients, as well as patients that suffered bilateral wrist fractures or other injuries to the affected limb are excluded. Patients that are not in the possession of a smartphone or tablet compatible with the serious game, as well as patients with a visual or mental impairment preventing the proper use of smartphone or tablet are excluded. Those unable to understand spoken or written Dutch or English will be unable to complete the questionnaires and are therefore not eligible for participation.

Table 1. Inclusion and exclusion criteria

Inclusion criteria	Exclusion criteria
1. Any type of closed distal radius fracture with no injury to the skin of the affected limb.<br><br>2. Good position after reposition or operative fixation, defined by <10 degrees of inclination in	1. Polytraumatized patients (Injury Severity Score ≥16).<br><br>2. Bilateral wrist fractures or other concomitant injuries to the affected limb.

For peer review only - http://bmjopen.bmj.com/site/about/guidelines.xhtml

any direction, <5 mm shortening of the radius compared to the ulna, <2 mm disposition of intra-articular fragments.	
3. Fracture primarily treated with conservative (cast) immobilization or operative fixation (ORIF).	3. Patients with other disease or injury causing a clinically relevant loss of function or range of motion in the wrist, as reported by patients (including Parkinson's disease, having had a cerebral vascular accident, amyotrophic lateral sclerosis, neuropathy of any kind).
4. Fracture considered to be consolidated by treating physician (trauma- or orthopaedic surgeon or surgical resident in training)	4. Previous fractures or any condition affecting the injured wrist with clinically relevant residual pain, loss of function or range of motion.
5. Possible to start rehabilitation exercises within 5 days after cast removal or operative fixation, as decided by treating physician (trauma- or orthopaedic surgeon or surgical resident in training)	5. Any medical contra-indication to start rehabilitation within 5 days after operation or cast removal, including dislocation of the fracture, tendon rupture or complex regional pain syndrome, as decided by the treating specialist.
6. Age ≥ 18 years.	6. Insufficient proficiency of Dutch or English in speech and written language, or inability to complete the Dutch questionnaires.
7. Written informed consent.	7. Not in the possession of, or able to obtain for the duration of our study, a smartphone or tablet compatible with the serious game.
	8. Visual impairment preventing use of the smartphone-based game.

Randomisation

When patients have been found eligible, and after they signed the informed consent form, baseline measurements are obtained. After baseline measurements, the local researcher contacts the central research coordinator for randomisation to take place, and to receive information on group allocation. Randomisation sequence is determined by an independent researcher using an online automatically generated randomisation list (via www.sealedenvelope.com). Allocation concealment is obtained using sealed opaque envelopes. Patients are randomised using block randomization in blocks of four, stratified for age (18-64, and 65 or above) and for treatment type (conservative treatment using immobilisation, or operative treatment by means of internal fixation) to ensure a balanced randomisation into the two groups.

For peer review only

For peer review only - http://bmjopen.bmj.com/site/about/guidelines.xhtml

Interventions

After randomisation, patients start participation in the trial as soon as their fracture has been qualified as stable or consolidated, and patients are fit to start active exercises as determined by the clinician; this is either directly, or as soon as possible, but within 3-5 days after cast removal or successful operative fixation. Together with specialized hand and wrist physiotherapists and exercise therapists, a standardized protocol for the first six weeks – when most progress is to be expected – has been established that is both embedded within the game and explained to patients.[38] All patients are instructed by their clinician to perform the hand and wrist rehabilitation exercises 3-5 times per day. In addition, patients receive a simple diary to complete, with instructions to register their frequency and duration of exercises daily, as well as any physical complaints they may have during exercising.

<u>Intervention group</u>

Patients that are randomized to the game group receive a download link via email, together with instructions on how to download and install the game on their own smartphone or tablet (iPad® or iPhone®, Apple Inc., Cupertino, CA, USA). Patients are instructed to strap one motion sensor to the dorsum of the hand, and to strap a second one to the proximal forearm (Figure 1). If patients are only in the possession of a compatible smartphone, they will receive the Myo™ gesture control armband (Thalmic Labs Inc. Kitchener, Ontario, Canada). This sensor is worn around the hand, in combination with the patient's own smartphone strapped around the forearm with the screen upwards and still visible (Figure 1). If patients are only in the possession of a compatible tablet, or if they prefer this option over using their smartphone, they receive two Valedo® sensors (Hocoma AG, Zurich, Switzerland). Two separate sensors are used since the tablet cannot be strapped to the arm to be used as a motion sensor. Both types of sensors control the exact same game in the same manner, the motions are the same, and gameplay in both types of game controller has been validated extensively, as described previously.[38] Both types of motion sensors are CE-marked, have been found fit to act

For peer review only - http://bmjopen.bmj.com/site/about/guidelines.xhtml

as a game controller and are able to monitor wrist range of motion. The sensors are connected to the smartphone via Bluetooth. By using two separate motion sensors placed proximally and distally of the wrist joint, isolated wrist motions are used for game control and patients cannot 'cheat' by moving the shoulder joint or the fingers. All otherwise necessary instructions are embedded within the game, making the game 'self-explanatory'. Intervention group patients will practice with the game for six weeks, after which they return the motion sensor to the hospital and are encouraged to continue exercising independently.

For peer review only

Gameplay

The *ReValidate!* game shows an underwater world in which the patient plays one level consisting of three different mini-games of similar difficulty (Supplementary video). Each mini-game has its own avatar, and is controlled by one specific wrist motion: the 'anglerfish' mini-game is controlled by pronation and supination, the 'shark' mini-game is controlled by palmar and dorsal flexion of the wrist, and the 'penguin' mini-game is controlled by radial an ulnar deviation (Figure 2). The patient plays one level 3-5 times per day, for a duration of approximately 10-15 minutes. Every playing session starts with a 'warm-up' of the motions, during which the motion sensors are calibrated and the game is set to the patients' own maximum range of motion. This ensures the game remains challenging yet playable, and prevents overstraining the wrist to make movements outside of the patients' own range of motion. A new level is unlocked each day, provided that at least one playing session has been completed that day, so the game remains motivating and challenging. All levels consist of an underwater parkour that needs to be completed, only the surroundings and route change. Levels gradually increase in length and difficulty over the course of six weeks, in the same manner as a physiotherapy treatment program increases in intensity over time. Patients are motivated by a daily 'push notification' reminder to exercise, and by optional high score rankings. Bonus points can be gathered by playing consistently 3-5 times per day and by obtaining 'collectibles'

For peer review only - http://bmjopen.bmj.com/site/about/guidelines.xhtml

found in the different levels. The game application registers frequency and duration of gameplay and records progress in terms of range of motion in degrees.

Because the swimming motions of the fish are comparable to the wrist motions needed to control the mini-games, gameplay through the embedded wrist exercises is intuitive and natural.

The game has been designed specifically for wrist rehabilitation by a company specialized in the development of rehabilitation games. An iterative design process was setup to ensure embedding of the standard exercise protocols. The game has been tested at all stages of development by experts and novices, including trauma surgeons, specialised hand and wrist surgeons, occupational therapists and physiotherapists, as well as by end-users.[38] Content has been checked for validity and exercise completeness both by user groups of patients and medical experts, and was found to be similar to regular physiotherapy exercise regimens.[38] Should patients encounter any problems with the game, they are provided contact details of a study coordinator. For any medical questions or concerns, patients are referred back to their treating specialist. Though not preferred, patients receive a referral for additional physiotherapy upon request. They will be motivated by their clinician to continue practicing using the game. The number of physiotherapy visits will be registered prospectively.

<u>Control group</u>

Patients in the control group receive standard care, consisting of home-based unsupervised wrist exercises, and upon request or recommendation by their clinician, a referral for specialized hand physiotherapy or occupational therapy. An overview of simple exercises for the three main motions in the wrist (palmar/dorsal flexion, pronation/supination and radial/ulnar deviation), is provided on paper, together with an explanation of exercises by the clinician. Patients are instructed to perform controlled movements up to a point that cause a stretch but no pain. Patients are instructed to practice these movements three to five times daily, approximately 10-15 minutes per session. This regimen has been developed in cooperation with specialized hand and wrist physiotherapists and

For peer review only - http://bmjopen.bmj.com/site/about/guidelines.xhtml

For peer review only

occupational therapists, and is most reflective of current standard practice. All patients referred to physiotherapy are instructed to perform active exercises without load. The number of physiotherapy visits will be registered prospectively.

Outcome measurements

Standard demographic information including age, sex, fracture type according to the AO/OTA (Arbeitsgemeinschaft für Osteosynthesefragen/Orthopaedic Trauma Association) fracture classification,[39] and the type of treatment is obtained directly after inclusion. Follow-up visits take place at two, six, and twelve weeks postoperatively or after cast removal; for operatively or conservatively treated patients, respectively. A timeline of the trial is shown in figure 3. The primary outcome measure, which is the validated patient-rated wrist evaluation (PRWE) questionnaire is completed by patients at all follow-up visits.[40,41] The active range of motion (ROM) is measured in degrees using an analogue universal goniometer. Range of motion (ROM) and grip strength are measured in both hands, where grip strength is measured in kilograms, using an analogue hand dynamometer (Baseline®, Fabrication Enterprises Inc., White Plains, NY, USA). The maximum value of two measurements with five minutes rest in between is used for analysis. All measurements are performed by a clinician blinded to group allocation that has been specifically trained for this trial, and is experienced in taking ROM and strength measurements in daily practice.

Patients in both study arms complete a questionnaire, that was specifically developed for this trial, on their self-efficacy, experiences and perceived effectiveness of their exercise program. Pain scores on a visual analogue scale (VAS) are recorded, and patients are asked about the frequency, if any, of visits to a physiotherapist or occupational therapist. In addition, partial or complete return to work, or to normal functioning if patients were currently unemployed, is registered. Patients are reminded by the researcher to fill out their exercise diary at every follow-up visit, and are asked to return their completed exercise diary at six weeks of follow-up. In the intervention group, the data on exercise frequency and duration, as well as range of motion, will also be retrieved from the serious game. The

For peer review only - http://bmjopen.bmj.com/site/about/guidelines.xhtml

frequency reported by patients in this group will be compared to the data from the game application, which stores all exercise data including frequency, duration and the range of motion measured.

Sample size calculation

The primary outcome is the PRWE questionnaire outcome score after 6 weeks of treatment. This questionnaire has been validated previously,[40] and has a minimal clinically important difference (MCID) of 11.5, with 1.5 for the pain subscale, and 10 for the function subscale.[41]

An elaborate and important Cochrane review on the effect of physiotherapy after distal radius fractures reports only three studies comparing a regular physiotherapy regimen to an unsupervised home-exercise program.[15] These comparisons are relevant here, since the use of this serious game can be compared to an un supervised home-exercise program. Only one of these three trials reports patient-rated functional outcomes in addition to range of motion or grip strength values.[18] This study looks into the effects of a progressive home-exercise program, compared to the effect of 12 physiotherapy sessions over a 6 week period in patients with an operatively treated distal radius fracture. They found a positive effect in favour of the home-exercise program, with PRWE scores of 18.5 (standard deviation 15.9) for the home-exercise group, compared to 36.1 (SD 13.9) in the physiotherapy group after 6 weeks.[18]

As a result, using the standard deviation of 15.9, for a two-sided t-test in two groups with an alpha of 0.05, a power of 90% (1 – beta = 0.9), an allocation ratio of 1:1, this leads to necessary group sizes of 42 patients per group to detect a clinically relevant difference of 11.5 points at six weeks. With a 10% dropout rate, a total of 92 patients are needed for this trial.

Data analysis

The primary outcome is the PRWE-score at six weeks. This outcome is represented as the change in outcome scores from T0 to T2 (delta, T2 – T0). An intention-to-treat analysis is performed, and any

For peer review only - http://bmjopen.bmj.com/site/about/guidelines.xhtml

For peer review only

missing data is imputed. Data will be analysed using a two-sided t-test or the Mann-Whitney U test, depending on normality of the distribution.

The secondary continuous outcomes (PRWE score at 2 and 12 weeks, ROM, grip strength, pain scores), a repeated measures analysis of variance will be performed, where group assignment (game group vs. control group) is the between-subjects factor, and time of visit (baseline, 2, 6 or 12 weeks follow-up) is the within-subjects factor. In case of a non-normal distribution, Generalised Estimating Equations (GEE) will be used. An intention-to-treat analysis will be performed, and any missing data will be imputed using multiple imputation. Other (secondary) functional outcomes are analysed as a percentage of the unaffected wrist. Bonferroni correction is applied to all secondary outcomes to correct for multiple tests. Therefore, P-values of < 0.0045 will be considered statistically significant. Baseline data and secondary outcomes include categorical data such as fracture type and treatment type. These data will be analysed using the Chi-square test, and outcomes will be presented as the differences in frequencies. P-values of < 0.05 are considered statistically significant. Treatment adherence is measured using a self-reported diary. The number of exercise sessions and the total duration of exercising are continuous variables that will undergo quantitative and qualitative synthesis using a two-sided t-test or a Mann-Whitney U test as appropriate.

The diaries that are handed in after the trial period are analysed for the percentage of days the diary is completed and the total estimated time of exercising. In the intervention group this data is also compared to the total time of exercising as registered within the game. In addition, should not all diaries be completed, the number of diaries handed in after the study period are registered. These data are then analysed using the Chi-square test, and outcomes will be presented as the difference in frequencies.

Patient and public involvement

Patients were involved during development, playtesting and previous validation of the game. No patients were involved in the design, recruitment or conduct of this study. Results of the study will

For peer review only - http://bmjopen.bmj.com/site/about/guidelines.xhtml

be published on the trial website, which is available publicly. The burden of the intervention has been evaluated by patients taking part in a specialised rehabilitation program at a hand and wrist physiotherapy practice. They considered playing the game to be a comparable burden to their prescribed exercises.

For peer review only

For peer review only - http://bmjopen.bmj.com/site/about/guidelines.xhtml

ETHICS AND DISSEMINATION

This study will be conducted according to the principles of the Declaration of Helsinki (64th WMA General Assembly, Fortaleza, Brazil, October 2013) and in accordance with the Medical Research Involving Human Subjects Act (WMO). The dataset will be made available and results of our study will be published unreservedly through academic journals, (international) conferences and popular media.

The medical risks of this trial are considered to be low, since treatment of both the intervention and the control group complies with national guidelines for standard treatment of distal radius fractures. Participation, or the choice to not participate, will not have any effect on any part of patient treatment or on the quality of medical care for patients.

Storing and processing of all patient data occurs in compliance with the General Data Protection Act (GDPR), and a data processing agreement has been established between the app manufacturer and the hospital leading the trial. Digital data gathered by the individual mobile applications has been assessed using a Data Privacy Impact Assessment (DPIA), and approved of by the hospitals' Data Privacy Officer (DPO). All digital data is stored on a computer server located within the highly secured hospital network and can only be accessed by the researchers after logging in with their personal account. The compliance with these rules ensures that privacy risks are minimised.

All data regarding the trial subjects is stored in a secure location in a locked cabinet that can only be accessed by study personnel. Data is anonymised and stored according to subject number. A linking log is stored separately from the data. All data, both digital and hardcopy, is stored for fifteen years after trial completion according to WMO regulations.

For peer review only

For peer review only - http://bmjopen.bmj.com/site/about/guidelines.xhtml

DISCUSSION

There is a rapid increase in the use of technological applications for healthcare support and patient self-management.[33,42,43] Clinical research on the effectiveness of these innovative applications is still scarce, however.[32] 'Wearables' show potential as monitoring devices, since they are non-obtrusive and can monitor patients over longer periods of time.[44] In addition, serious games have shown their own potential in education, improving both motivation and learning outcomes,[45,46] and have also shown to be promising tools to increase self-efficacy; an important factor contributing to treatment adherence and physiotherapy outcomes.[28,30]

Readily available computer games using body motion for control, for example the Nintendo® Wii™, have already been researched in clinical settings as treatment support tools.[33] Though, the issue with off-the-shelf games is that they are not designed as a medical device, hence lack proper validation for medical use. They may increase activity and motivation in a rehabilitation process, yet have been designed for entertainment purposes.[38] Furthermore, these games usually require larger consoles that limit the patient in performing the exercises anywhere they want.

To the authors' knowledge, this study is the first study focusing on a validated, wearable-controlled serious game designed to act as a medical support tool for wrist rehabilitation. This game and similar interventions may decrease the ever-growing burden wrist injuries pose to patients and to society, and can make validated wrist exercise therapy easily accessible for anyone. This study will contribute to the advancements of serious games for traumatic and non-traumatic wrist and other injuries, and may pave the way for future development and validation of wearable-controlled rehabilitation games.

For peer review only - http://bmjopen.bmj.com/site/about/guidelines.xhtml

ACKNOWLEDGEMENTS

We acknowledge all volunteers and patient advisers, physiotherapists and hand therapists that have collaborated during development of the game. We especially thank A.J. Videler of the Hand- and Wrist Centre, Amsterdam, and C.J.M. Nooij of the department of Rehabilitation, Amsterdam UMC, location Academic Medical Centre. We thank Motek Medical B.V. and Virtual Play for the collaborative development of the game. Furthermore, we thank all supporting physicians, specialised nurses and other nurses, and outpatient clinic staff from all collaborating hospitals that made the realization of this trial possible.

FOOTNOTES

Author contributions:

All authors have contributed to the design of this trial protocol. MPS and JCG initiated the project. The protocol was drafted by HAWM, and revised by MCO, MG, MPS, SVD and JCG. SVD provided statistical and methodological support. All authors have read and approved the final manuscript. The ReValidate! collaborative study group consists of local investigators who are responsible for trial execution, patient inclusion and data collection. They have all read and approved the final manuscript.

Conflict of interest statement:

The authors have no conflicts of interest to declare.

Funding:

The ReValidate! project received funding by the Growing Games Program (Dutch Game Garden, iMMovator, Dutch Games Association and the Economic Board Utrecht), grant number CB00018, by a grant from the CZ Fund, the Netherlands. The sponsor had no role of any sort in the study design.

For peer review only - http://bmjopen.bmj.com/site/about/guidelines.xhtml

Patient consent:

Obtained

Ethics approval:

This study has been evaluated and approved by the Medical Ethical Review Board at the Academic Medical Centre, Amsterdam, the Netherlands.

Collaborators:

Prof. P. Kloen, MD PhD, Amsterdam UMC, location Academic Medical Centre, Amsterdam, the Netherlands;

H.W. Bolhuis, MD PhD, Gelre ziekenhuizen, Apeldoorn, the Netherlands;

S.R. de Wild, MD, Gelre Ziekenhuizen, Apeldoorn, the Netherlands;

T. Herklots, MD, Gelre Ziekenhuizen, Apeldoorn, the Netherlands;

J. Baaij, MD MSc, Gelre Ziekenhuizen, Apeldoorn, the Netherlands;

J. Winkelhagen, MD, Dijklanderziekenhuis, Hoorn, the Netherlands;

R. Buijsman, MD, Amsterdam UMC, location Academic Medical Centre, Amsterdam, the Netherlands;

K.T. van Hamersveld, MD, Dijklanderziekenhuis, Hoorn, the Netherlands;

B.A. van Dijkman, MD PhD, Flevoziekenhuis, Almere, the Netherlands;

M. Jansen, MD, Amsterdam UMC, location Academic Medical Centre, Amsterdam, the Netherlands;

E.Z. Barsom, MD, Amsterdam UMC, location Academic Medical Centre, Amsterdam, the Netherlands;

S.L. van der Storm, MD, Amsterdam UMC, location Academic Medical Centre, Amsterdam, the Netherlands;

N. van Oorschot, MD, Amsterdam UMC, location Academic Medical Centre, Amsterdam, the Netherlands;

M.J. van der Pols, Amsterdam UMC, location Academic Medical Centre, Amsterdam, the Netherlands.

For peer review only - http://bmjopen.bmj.com/site/about/guidelines.xhtml

REFERENCES

1. Mellstrand-Navarro C, Pettersson HJ, Tornqvist H, et al. The operative treatment of fractures of the distal radius is increasing: results from a nationwide Swedish study. *The bone & joint journal* 2014;96-B(7):963-9. doi: 10.1302/0301-620X.96B7.33149 [published Online First: 2014/07/06]
2. Court-Brown CM, Caesar B. Epidemiology of adult fractures: A review. *Injury* 2006;37(8):691-7. doi: 10.1016/j.injury.2006.04.130 [published Online First: 2006/07/04]
3. Kilgore ML, Morrisey MA, Becker DJ, et al. Health care expenditures associated with skeletal fractures among Medicare beneficiaries, 1999-2005. *J Bone Miner Res* 2009;24(12):2050-5. doi: 10.1359/jbmr.090523 [published Online First: 2009/05/21]
4. Nellans KW, Kowalski E, Chung KC. The epidemiology of distal radius fractures. *Hand Clin* 2012;28(2):113-25. doi: 10.1016/j.hcl.2012.02.001 [published Online First: 2012/05/05]
5. Karl JW, Olson PR, Rosenwasser MP. The Epidemiology of Upper Extremity Fractures in the United States, 2009. *J Orthop Trauma* 2015;29(8):e242-4. doi: 10.1097/BOT.0000000000000312 [published Online First: 2015/02/26]
6. MacIntyre NJ, Dewan N. Epidemiology of distal radius fractures and factors predicting risk and prognosis. *J Hand Ther* 2016;29(2):136-45. doi: 10.1016/j.jht.2016.03.003 [published Online First: 2016/06/07]
7. Chung KC, Spilson SV. The frequency and epidemiology of hand and forearm fractures in the United States. *J Hand Surg Am* 2001;26(5):908-15. doi: 10.1053/jhsu.2001.26322 [published Online First: 2001/09/19]
8. Brogren E, Petranek M, Atroshi I. Incidence and characteristics of distal radius fractures in a southern Swedish region. *BMC Musculoskelet Disord* 2007;8:48. doi: 10.1186/1471-2474-8-48 [published Online First: 2007/06/02]
9. Stirling ERB, Johnson NA, Dias JJ. Epidemiology of distal radius fractures in a geographically defined adult population. *Journal of Hand Surgery (European Volume)* 2018;43(9):974-82. doi: 10.1177/1753193418786378
10. Angermann P, Lohmann M. Injuries to the hand and wrist. A study of 50,272 injuries. *J Hand Surg Br* 1993;18(5):642-4. doi: 10.1016/0266-7681(93)90024-a [published Online First: 1993/10/01]
11. de Putter CE, Selles RW, Polinder S, et al. Economic impact of hand and wrist injuries: health-care costs and productivity costs in a population-based study. *J Bone Joint Surg Am* 2012;94(9):e56. doi: 10.2106/JBJS.K.00561 [published Online First: 2012/05/04]
12. Larsen CF, Mulder S, Johansen AMT, et al. The epidemiology of hand injuries in the Netherlands and Denmark. *Eur J Epidemiol* 2004;19(4):323-27.
13. Bonafede M, Espindle D, Bower AG. The direct and indirect costs of long bone fractures in a working age US population. *J Med Econ* 2013;16(1):169-78. doi: 10.3111/13696998.2012.737391 [published Online First: 2012/10/06]
14. Fricker R, Jupiter J, Kastelec M. AO Surgery reference: Distal forearm [Web page]. Davos, Switzerland: AO Foundation; 2017 [Available from: https://www2.aofoundation.org/wps/portal/surgery?showPage=diagnosis&bone=Radius&segment=Distal accessed Jun 4 2017.
15. Handoll HHG, Elliott J. Rehabilitation for distal radial fractures in adults (Cochrane review) [with consumer summary]. *Cochrane Database of Systematic Reviews 2005;Issue 9* 2015
16. Bruder AM, Shields N, Dodd KJ, et al. A progressive exercise and structured advice program does not improve activity more than structured advice alone following a distal radial fracture: a multi-centre, randomised trial. *Journal of physiotherapy* 2016;62(3):145-52. doi: 10.1016/j.jphys.2016.05.011
17. Gutierrez-Espinoza H, Rubio-Oyarzun D, Olguin-Huerta C, et al. Supervised physical therapy vs home exercise program for patients with distal radius fracture: A single-blind randomized clinical study. *Journal of Hand Therapy* 2017;07 doi: 10.1016/j.jht.2017.02.001

For peer review only - http://bmjopen.bmj.com/site/about/guidelines.xhtml

18. Krischak G, Krasteva A, Schneider F, et al. Physiotherapy after volar plating of wrist fractures is effective using a home exercise program. *Archives of physical medicine and rehabilitation* 2009; 90(4).
19. Brehmer JL, Husband JB. Accelerated rehabilitation compared with a standard protocol after distal radial fractures treated with volar open reduction and internal fixation: A prospective, randomized, controlled study. *Journal of Bone and Joint Surgery - American Volume* 2014;96(19):1621-30. doi: 10.2106/JBJS.M.00860
20. Lozano-Calderón S, Souer S, Mudgal C, et al. Wrist mobilization following volar plate fixation of fractures of the distal part of the radius. *The Journal of bone and joint surgery American volume* 2008; 90(6).
21. Kay S, McMahon M, Stiller K. An advice and exercise program has some benefits over natural recovery after distal radius fracture: a randomised trial. *The Australian journal of physiotherapy* 2008; 54(4).
22. American Academy of Orthopaedic Surgeons. The treatment of distal radius fractures - Guideline and evidence report. Recommendation 21-23. Rosemont, IL: AAOS, 2009:84-92.
23. Deutschen Gesellschaft für Unfallchirurgie e.V. (DGU). Distale Radiusfraktur - Leitlinie Unfallchirurgie. Physiotherapie. Göttingen: DGU, 2014:32-33.
24. British Orthopaedic Association, British Society for Surgery of the Hand. Best practice for management of Distal Radial Fractures (DRFs). Type of rehailitation intervention, mode of delivery and discipline of deliverer. London, UK, 2018:107.
25. Nederlandse Vereniging voor Heelkunde. Richtlijn Distale Radiusfracturen: diagnostiek en behandeling., 2010.
26. Sluijs EM, Kok GJ, van der Zee J. Correlates of exercise compliance in physical therapy. *Phys Ther* 1993;73(11):771-82; discussion 83-6. doi: 10.1093/ptj/73.11.771 [published Online First: 1993/11/01]
27. Martin LR, Williams SL, Haskard KB, et al. The challenge of patient adherence. *Ther Clin Risk Manag* 2005;1(3):189-99. [published Online First: 2008/03/25]
28. Picha KJ, Howell DM. A model to increase rehabilitation adherence to home exercise programmes in patients with varying levels of self-efficacy. *Musculoskeletal Care* 2018;16(1):233-37. doi: 10.1002/msc.1194 [published Online First: 2017/04/13]
29. Lyngcoln A, Taylor N, Pizzari T, et al. The relationship between adherence to hand therapy and short-term outcome after distal radius fracture. *J Hand Ther* 2005;18(1):2-8; quiz 9. doi: 10.1197/j.jht.2004.10.008 [published Online First: 2005/01/28]
30. Bjork M, Niklasson J, Westerdahl E, et al. Self-efficacy corresponds to wrist function after combined plating of distal radius fractures. *J Hand Ther* 2020 doi: 10.1016/j.jht.2020.01.001 [published Online First: 2020/02/24]
31. Burke JW, McNeill MDJ, Charles DK, et al. Optimising engagement for stroke rehabilitation using serious games. *Visual Comput* 2009;25(12):1085-99. doi: 10.1007/s00371-009-0387-4
32. Meijer HA, Graafland M, Goslings JC, et al. Systematic Review on the Effects of Serious Games and Wearable Technology Used in Rehabilitation of Patients With Traumatic Bone and Soft Tissue Injuries. *Arch Phys Med Rehabil* 2018;99(9):1890-99. doi: 10.1016/j.apmr.2017.10.018 [published Online First: 2017/11/16]
33. Laver KE, Lange B, George S, et al. Virtual reality for stroke rehabilitation. *Cochrane Database Syst Rev* 2017;11:CD008349. doi: 10.1002/14651858.CD008349.pub4 [published Online First: 2017/11/21]
34. Bergeron BP. Developing Serious Games. 1st ed. Hingham, MA: Charles River Media 2006.
35. Brichetto G, Spallarossa P, de Carvalho ML, et al. The effect of Nintendo(R) Wii(R) on balance in people with multiple sclerosis: a pilot randomized control study. *Multiple sclerosis (Houndmills, Basingstoke, England)* 2013;19(9):1219-21. doi: 10.1177/1352458512472747 [published Online First: 2013/01/17]
36. Butler DP, Willett K. Wii-habilitation: is there a role in trauma? *Injury* 2010;41(9):883-5. doi: 10.1016/j.injury.2010.03.024 [published Online First: 2010/04/20]

For peer review only - http://bmjopen.bmj.com/site/about/guidelines.xhtml

37. Morone G, Tramontano M, Iosa M, et al. The efficacy of balance training with video game-based therapy in subacute stroke patients: a randomized controlled trial. *BioMed research international* 2014;2014:580861. doi: 10.1155/2014/580861 [published Online First: 2014/05/31]
38. Meijer HAW, Graafland M, Obdeijn MC, et al. Face Validity and Content Validity of a Game for Distal Radius Fracture Rehabilitation. *J Wrist Surg* 2019;8(5):388-94. doi: 10.1055/s-0039-1688948 [published Online First: 2019/10/04]
39. Meinberg EG, Agel J, Roberts CS, et al. Fracture and Dislocation Classification Compendium-2018. *J Orthop Trauma* 2018;32 Suppl 1:S1-S170. doi: 10.1097/BOT.0000000000001063 [published Online First: 2017/12/20]
40. MacDermid JC, Tottenham V. Responsiveness of the disability of the arm, shoulder, and hand (DASH) and patient-rated wrist/hand evaluation (PRWHE) in evaluating change after hand therapy. *J Hand Ther* 2004;17(1):18-23. doi: 10.1197/j.jht.2003.10.003 [published Online First: 2004/02/11]
41. Walenkamp MM, de Muinck Keizer RJ, Goslings JC, et al. The Minimum Clinically Important Difference of the Patient-rated Wrist Evaluation Score for Patients With Distal Radius Fractures. *Clin Orthop Relat Res* 2015;473(10):3235-41. doi: 10.1007/s11999-015-4376-9
42. Arrebola LS, Yi LC, De Oliveira VGC. The use of video games combined with conventional physical therapy in children with upper limb fractures: An exploratory study. *Journal of Pediatric Rehabilitation Medicine* 2019;12(1):65-70. doi: 10.3233/PRM-170529
43. Bruder AM, McClelland JA, Shields N, et al. Validity and reliability of an activity monitor to quantify arm movements and activity in adults following distal radius fracture. *Disability & Rehabilitation* 2018;40(11):1318-25. doi: 10.1080/09638288.2017.1288764
44. Bonato P. Wearable sensors/systems and their impact on biomedical engineering. *IEEE engineering in medicine and biology magazine : the quarterly magazine of the Engineering in Medicine & Biology Society* 2003;22(3):18-20.
45. Graafland M, Bemelman WA, Schijven MP. Appraisal of face and content validity of a serious game improving situational awareness in surgical training. *Journal of laparoendoscopic & advanced surgical techniques Part A* 2015;25(1):43-9. doi: 10.1089/lap.2014.0043
46. Graafland M, Vollebergh MF, Lagarde SM, et al. A serious game can be a valid method to train clinical decision-making in surgery. *World journal of surgery* 2014;38(12):3056-62. doi: 10.1007/s00268-014-2743-4

For peer review only

For peer review only - http://bmjopen.bmj.com/site/about/guidelines.xhtml

Figure legends

Figure 1: Sensor placement. A: iPhone® and Myo™; B: two Valedo™ sensors.

Figure 2: Different avatars are used in the game. A: the anglerfish is controlled by pronation and supination; B: the shark is controlled by palmar and dorsal flexion; C: the penguin is controlled by radial and ulnar deviation.

Figure 3: Schematic timeline of the study showing the randomisation and follow-up planning, as well as the outcomes measured during each visit.

For peer review only

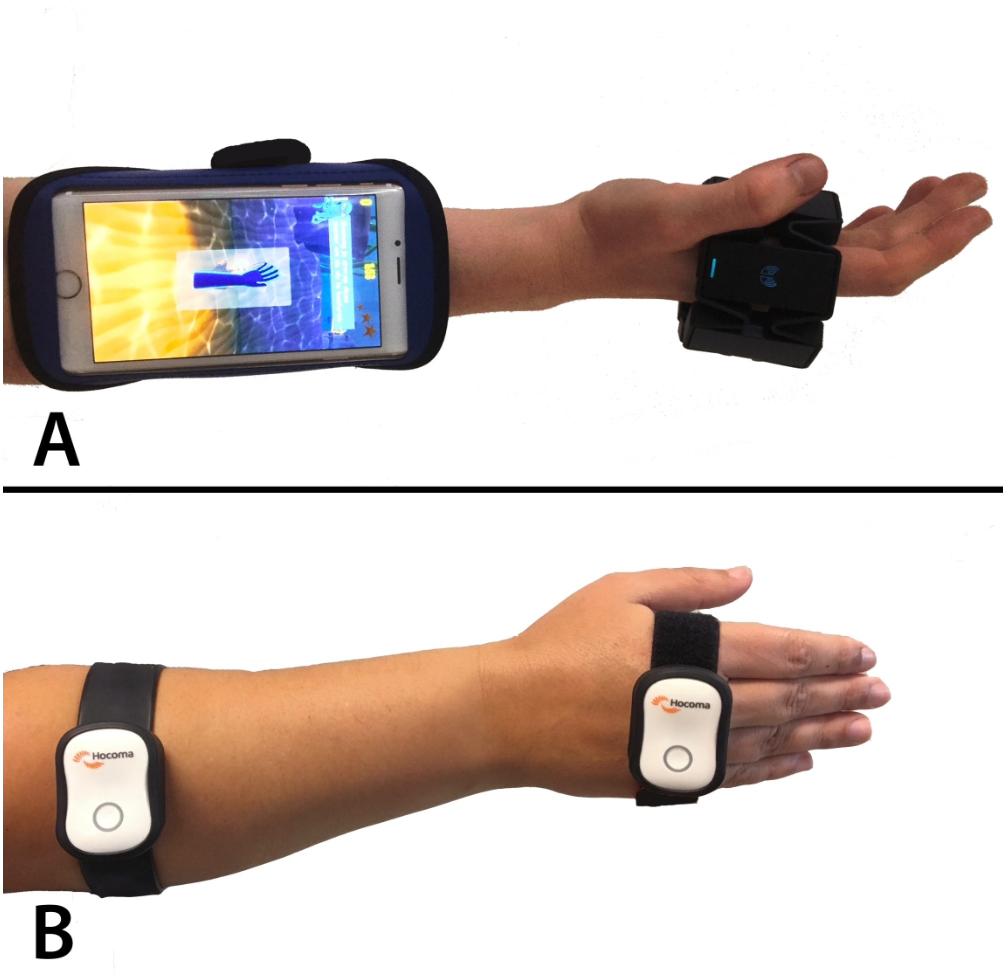

Figure 1: Sensor placement. A: iPhone® and Myo™; B: two Valedo™ sensors.

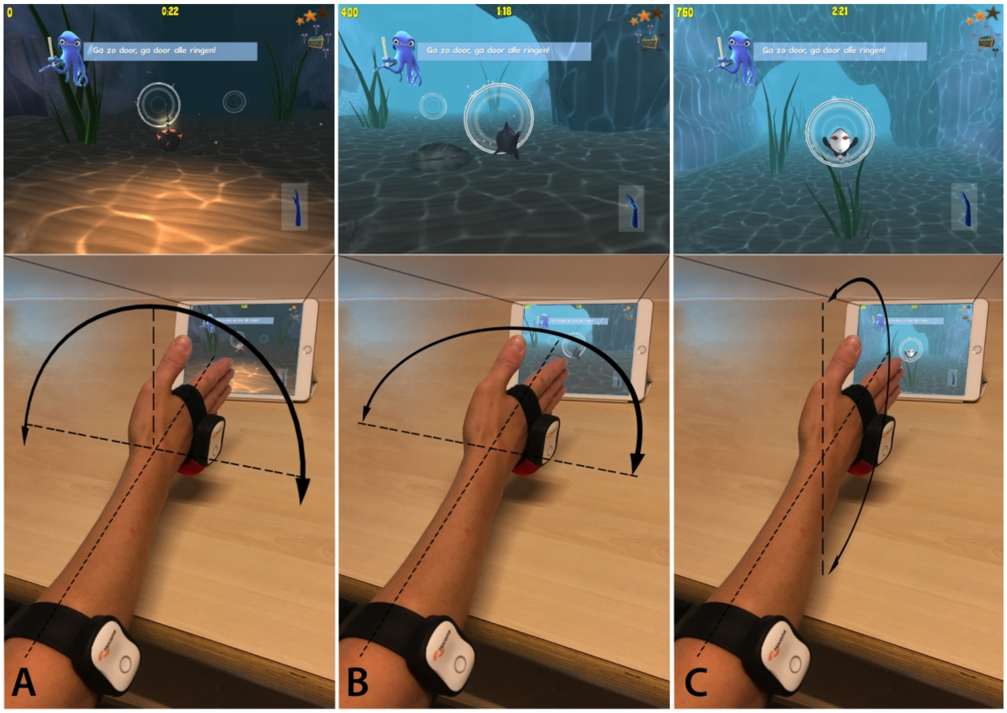

Figure 2: Different avatars are used in the game. A: the anglerfish is controlled by pronation and supination; B: the shark is controlled by palmar and dorsal flexion; C: the penguin is controlled by radial and ulnar deviation.

Figure 3: Schematic timeline

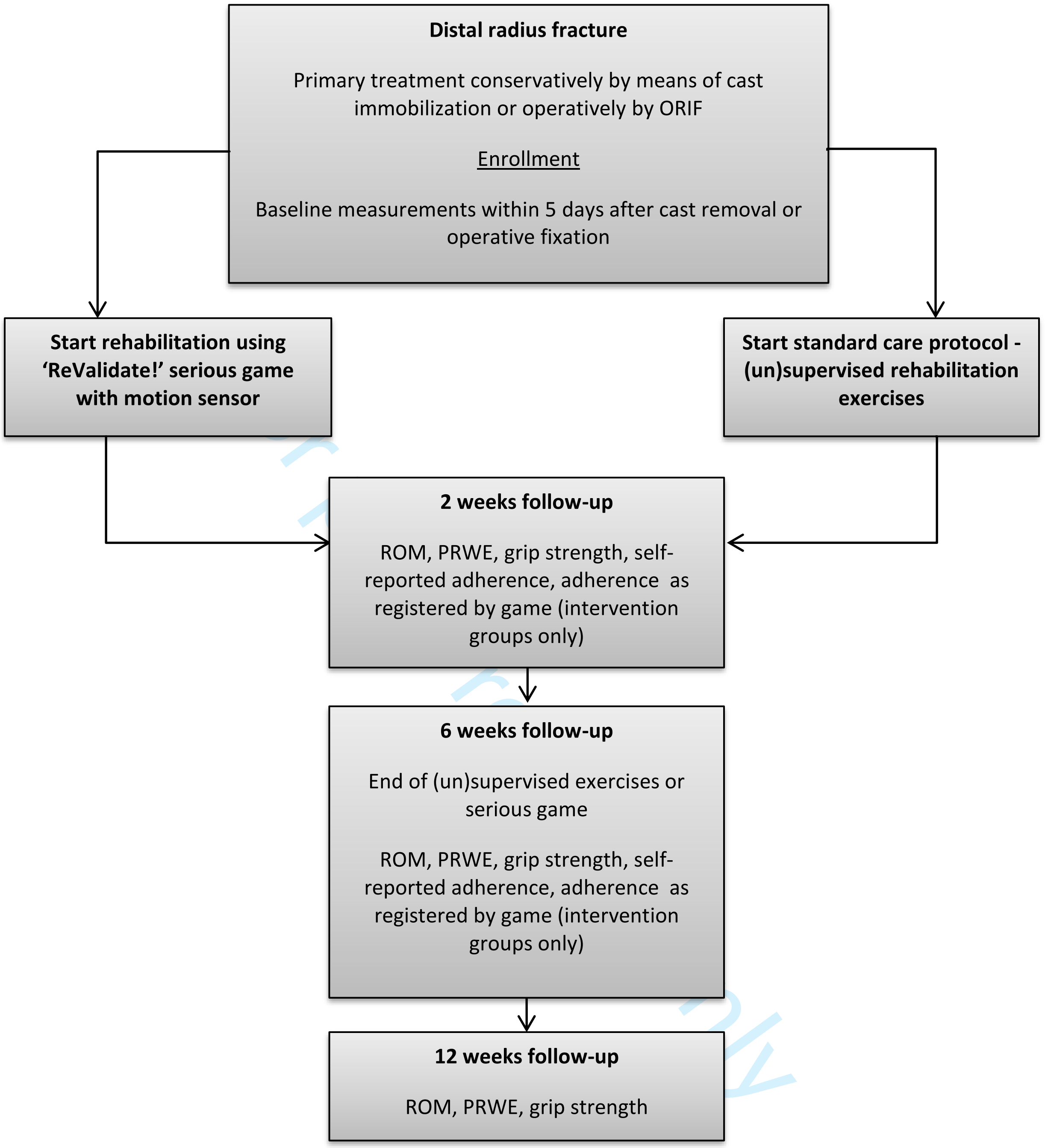

STANDARD PROTOCOL ITEMS: RECOMMENDATIONS FOR INTERVENTIONAL TRIALS

SPIRIT 2013 Checklist: Recommended items to address in a clinical trial protocol and related documents*

Section/item	Item No	Description	Addressed on page number
**Administrative information**			
Title	1	Descriptive title identifying the study design, population, interventions, and, if applicable, trial acronym	1 (both versions)
Trial registration	2a	Trial identifier and registry name. If not yet registered, name of intended registry	3 (clean version), 4 (marked version)
	2b	All items from the World Health Organization Trial Registration Data Set	3 (clean version), 4 (marked version)
Protocol version	3	Date and version identifier	3 (clean version), 4 (marked version)
Funding	4	Sources and types of financial, material, and other support	18 (clean version), 19 (marked version)
Roles and responsibilities	5a	Names, affiliations, and roles of protocol contributors	1, 18 (clean version), 1, 19 (marked version)
	5b	Name and contact information for the trial sponsor	1 (both versions)
	5c	Role of study sponsor and funders, if any, in study design; collection, management, analysis, and interpretation of data; writing of the report; and the decision to submit the report for publication, including whether they will have ultimate authority over any of these activities	18 (clean version), 19 (marked version)
	5d	Composition, roles, and responsibilities of the coordinating centre, steering committee, endpoint adjudication committee, data management team, and other individuals or groups overseeing the trial, if applicable (see Item 21a for data monitoring committee)	18 (clean version), 19 (marked version)

For peer review only

Introduction

Background and rationale	6a	Description of research question and justification for undertaking the trial, including summary of relevant studies (published and unpublished) examining benefits and harms for each intervention	4-6 (clean version), 5-6 (marked version)
	6b	Explanation for choice of comparators	4-5 (clean version), 5-6 (marked version)
Objectives	7	Specific objectives or hypotheses	5-6 (clean version), 6-7 (marked version)
Trial design	8	Description of trial design including type of trial (eg, parallel group, crossover, factorial, single group), allocation ratio, and framework (eg, superiority, equivalence, noninferiority, exploratory)	7 (clean version), 8 (marked version)

Methods: Participants, interventions, and outcomes

Study setting	9	Description of study settings (eg, community clinic, academic hospital) and list of countries where data will be collected. Reference to where list of study sites can be obtained	7 (clean version), 8 (marked version)
Eligibility criteria	10	Inclusion and exclusion criteria for participants. If applicable, eligibility criteria for study centres and individuals who will perform the interventions (eg, surgeons, psychotherapists)	7-8 (clean version), 8-9 (marked version) table 1
Interventions	11a	Interventions for each group with sufficient detail to allow replication, including how and when they will be administered	9-12 (clean version), 10-13 (marked version)
	11b	Criteria for discontinuing or modifying allocated interventions for a given trial participant (eg, drug dose change in response to harms, participant request, or improving/worsening disease)	10 (clean version), 11 (marked version)
	11c	Strategies to improve adherence to intervention protocols, and any procedures for monitoring adherence (eg, drug tablet return, laboratory tests)	12 (clean version), 13 (marked version)
	11d	Relevant concomitant care and interventions that are permitted or prohibited during the trial	11 (clean), 12 (marked)
Outcomes	12	Primary, secondary, and other outcomes, including the specific measurement variable (eg, systolic blood pressure), analysis metric (eg, change from baseline, final value, time to event), method of aggregation (eg, median, proportion), and time point for each outcome. Explanation of the clinical relevance of chosen efficacy and harm outcomes is strongly recommended	12-13 (clean), 13-14 (marked)
Participant timeline	13	Time schedule of enrolment, interventions (including any run-ins and washouts), assessments, and visits for participants. A schematic diagram is highly recommended (see Figure)	9, 12 (clean), 10, 13 (marked) Figure 3

For peer review only - http://bmjopen.bmj.com/site/about/guidelines.xhtml

Sample size	14	Estimated number of participants needed to achieve study objectives and how it was determined, including clinical and statistical assumptions supporting any sample size calculations	13 (clean), 14 (marked)
Recruitment	15	Strategies for achieving adequate participant enrolment to reach target sample size	7 (clean), 8 (marked)

Methods: Assignment of interventions (for controlled trials)

Allocation:

Sequence generation	16a	Method of generating the allocation sequence (eg, computer-generated random numbers), and list of any factors for stratification. To reduce predictability of a random sequence, details of any planned restriction (eg, blocking) should be provided in a separate document that is unavailable to those who enrol participants or assign interventions	8 (clean), 9 (marked)
Allocation concealment mechanism	16b	Mechanism of implementing the allocation sequence (eg, central telephone; sequentially numbered, opaque, sealed envelopes), describing any steps to conceal the sequence until interventions are assigned	8 (clean), 9(marked)
Implementation	16c	Who will generate the allocation sequence, who will enrol participants, and who will assign participants to interventions	8 (clean), 9(marked)
Blinding (masking)	17a	Who will be blinded after assignment to interventions (eg, trial participants, care providers, outcome assessors, data analysts), and how	12 (clean), 13 (marked)
	17b	If blinded, circumstances under which unblinding is permissible, and procedure for revealing a participant's allocated intervention during the trial	not applicable

Methods: Data collection, management, and analysis

Data collection methods	18a	Plans for assessment and collection of outcome, baseline, and other trial data, including any related processes to promote data quality (eg, duplicate measurements, training of assessors) and a description of study instruments (eg, questionnaires, laboratory tests) along with their reliability and validity, if known. Reference to where data collection forms can be found, if not in the protocol	12-13 (clean) 13-14 (marked)
	18b	Plans to promote participant retention and complete follow-up, including list of any outcome data to be collected for participants who discontinue or deviate from intervention protocols	11 (clean), 12 (marked)

Data management	19	Plans for data entry, coding, security, and storage, including any related processes to promote data quality (eg, double data entry; range checks for data values). Reference to where details of data management procedures can be found, if not in the protocol	16 (clean), 17 (marked)
Statistical methods	20a	Statistical methods for analysing primary and secondary outcomes. Reference to where other details of the statistical analysis plan can be found, if not in the protocol	13-14 (clean), 14-16 (marked)
	20b	Methods for any additional analyses (eg, subgroup and adjusted analyses)	13-14 (clean), 14-16 (marked)
	20c	Definition of analysis population relating to protocol non-adherence (eg, as randomised analysis), and any statistical methods to handle missing data (eg, multiple imputation)	13-14 (clean), 14-16 (marked)
**Methods: Monitoring**			
Data monitoring	21a	Composition of data monitoring committee (DMC); summary of its role and reporting structure; statement of whether it is independent from the sponsor and competing interests; and reference to where further details about its charter can be found, if not in the protocol. Alternatively, an explanation of why a DMC is not needed	not applicable
	21b	Description of any interim analyses and stopping guidelines, including who will have access to these interim results and make the final decision to terminate the trial	no interim analysis performed
Harms	22	Plans for collecting, assessing, reporting, and managing solicited and spontaneously reported adverse events and other unintended effects of trial interventions or trial conduct	not specified
Auditing	23	Frequency and procedures for auditing trial conduct, if any, and whether the process will be independent from investigators and the sponsor	not specified
**Ethics and dissemination**			
Research ethics approval	24	Plans for seeking research ethics committee/institutional review board (REC/IRB) approval	3, 19 (clean), 3, 20 (marked)
Protocol amendments	25	Plans for communicating important protocol modifications (eg, changes to eligibility criteria, outcomes, analyses) to relevant parties (eg, investigators, REC/IRBs, trial participants, trial registries, journals, regulators)	not specified

For peer review only - http://bmjopen.bmj.com/site/about/guidelines.xhtml

Consent or assent	26a	Who will obtain informed consent or assent from potential trial participants or authorised surrogates, and how (see Item 32)	9, not otherwise specified
	26b	Additional consent provisions for collection and use of participant data and biological specimens in ancillary studies, if applicable	not applicable
Confidentiality	27	How personal information about potential and enrolled participants will be collected, shared, and maintained in order to protect confidentiality before, during, and after the trial	16 (clean), 17 (marked)
Declaration of interests	28	Financial and other competing interests for principal investigators for the overall trial and each study site	18 (clean), 19 (marked)
Access to data	29	Statement of who will have access to the final trial dataset, and disclosure of contractual agreements that limit such access for investigators	16 (clean), 17 (marked)
Ancillary and post-trial care	30	Provisions, if any, for ancillary and post-trial care, and for compensation to those who suffer harm from trial participation	not applicable
Dissemination policy	31a	Plans for investigators and sponsor to communicate trial results to participants, healthcare professionals, the public, and other relevant groups (eg, via publication, reporting in results databases, or other data sharing arrangements), including any publication restrictions	3, 16 (clean), 3, 17 (marked)
	31b	Authorship eligibility guidelines and any intended use of professional writers	not specified
	31c	Plans, if any, for granting public access to the full protocol, participant-level dataset, and statistical code	16 (clean), 17 (marked)
**Appendices**			
Informed consent materials	32	Model consent form and other related documentation given to participants and authorised surrogates	attached as appendix
Biological specimens	33	Plans for collection, laboratory evaluation, and storage of biological specimens for genetic or molecular analysis in the current trial and for future use in ancillary studies, if applicable	not applicable

*It is strongly recommended that this checklist be read in conjunction with the SPIRIT 2013 Explanation & Elaboration for important clarification on the items. Amendments to the protocol should be tracked and dated. The SPIRIT checklist is copyrighted by the SPIRIT Group under the Creative Commons "Attribution-NonCommercial-NoDerivs 3.0 Unported" license.

For peer review only
